# Spatiotemporal regulation of ATP and Ca^2+^ dynamics in vertebrate rod and cone ribbon synapses

**Published:** 2007-06-15

**Authors:** Jerry E. Johnson, Guy A. Perkins, Anand Giddabasappa, Shawntay Chaney, Weimin Xiao, Andrew D. White, Joshua M. Brown, Jenna Waggoner, Mark H. Ellisman, Donald A. Fox

**Affiliations:** 1 Department of Natural Sciences, University of Houston-Downtown, Houston, TX; 2 College of Optometry, University of Houston, Houston, TX; 3 National Center for Microscopy and Imaging Research, University of California San Diego, La Jolla, CA; 4 Department of Biology and Biochemistry, University of Houston, Houston, TX; 5 Department of Neurosciences, University of California San Diego, La Jolla, CA; 6 Department of Pharmacology and Pharmaceutical Sciences, University of Houston, Houston, TX

## Abstract

**Purpose:**

In conventional neurons, Ca^2+^ enters presynaptic terminals during an action potential and its increased local concentration triggers transient exocytosis. In contrast, vertebrate photoreceptors are nonspiking neurons that maintain sustained depolarization and neurotransmitter release from ribbon synapses in darkness and produce light-dependent graded hyperpolarizing responses. Rods transmit single photon responses with high fidelity, whereas cones are less sensitive and exhibit faster response kinetics. These differences are likely due to variations in presynaptic Ca^2+^ dynamics. Metabolic coupling and cross-talk between mitochondria, endoplasmic reticulum (ER), plasma membrane Ca^2+^ ATPase (PMCA), and Na^+^-Ca^2+^ exchanger (NCX) coordinately control presynaptic ATP production and Ca^2+^ dynamics. The goal of our structural and functional studies was to determine the spatiotemporal regulation of ATP and Ca^2+^ dynamics in rod spherules and cone pedicles.

**Methods:**

Central retina tissue from C57BL/6 mice was used. Laser scanning confocal microscopy (LSCM) experiments were conducted on fixed-frozen vertical sections. Primary antibodies were selected for their tissue/cellular specificity and ability to recognize single, multiple or all splice variants of selected isoforms. Electron microscopy (EM) and 3-D electron tomography (ET) studies used our standard procedures on thin- and thick-sectioned retinas, respectively. Calibrated fluo-3-Ca^2+^ imaging experiments of dark- and light-adapted rod and cone terminals in retinal slices were conducted.

**Results:**

Confocal microscopy showed that mitochondria, ER, PMCA, and NCX1 exhibited distinct retinal lamination patterns and differential distribution in photoreceptor synapses. Antibodies for three distinct mitochondrial compartments differentially labeled retinal areas with high metabolic demand: rod and cone inner segments, previously undescribed cone juxtanuclear mitochondria and the two plexiform layers. Rod spherule membranes uniformly and intensely stained for PMCA, whereas the larger cone pedicles preferentially stained for NCX1 at their active zones and PMCA near their mitochondria. EM and ET revealed that mitochondria in rod spherules and cone pedicles differed markedly in their number, location, size, volume, and total cristae surface area, and cristae junction diameter. Rod spherules had one large ovoid mitochondrion located near its active zone, whereas cone pedicles averaged five medium-sized mitochondria clustered far from their active zones. Most spherules had one ribbon synapse, whereas pedicles contained numerous ribbon synapses. Fluo-3 imaging studies revealed that during darkness rod spherules maintained a lower [Ca^2+^] than cone pedicles, whereas during light adaptation pedicles rapidly lowered their [Ca^2+^] below that observed in spherules.

**Conclusions:**

These findings indicate that ATP demand and mitochondrial ATP production are greater in cone pedicles than rod spherules. Rod spherules employ high affinity/low turnover PMCA and their mitochondrion to maintain a relatively low [Ca^2+^] in darkness, which increases their sensitivity and signal-to-noise ratio. In contrast, cone pedicles utilize low affinity/high turnover NCX to rapidly lower their high [Ca^2+^] during light adaptation, which increases their response kinetics. Spatiotemporal fluo-3-Ca^2+^ imaging results support our immunocytochemical results. The clustering of cone pedicle mitochondria likely provides increased protection from Ca^2+^ overload and permeability transition. In summary, these novel studies reveal that several integrated cellular and subcellular components interact to regulate ATP and Ca^2+^ dynamics in rod and cone synaptic terminals. These results should provide a greater understanding of in vivo photoreceptor synaptic terminal exocytosis/endocytosis, Ca^2+^ overload and therapies for retinal degenerations.

## Introduction

During an action potential, Ca^2+^ enters the presynaptic terminal of conventional neurons through voltage-gated channels that cluster near the synaptic vesicle release sites or active zones. The increased subplasmalemmal Ca^2+^ concentration in these microdomains plays a key role in transient exocytosis [1-5]. Metabolic coupling and cross-talk between mitochondria, endoplasmic reticulum (ER), the plasma membrane Ca^2+^ ATPase (PMCA), and Na^+^-Ca^2+^ exchanger (NCX), and Ca^2+^ channels must coordinately control ATP production and Ca^2+^ dynamics in the presynaptic terminals to ensure recovery for the next action potential [1,2,4-9]. Thus, the spatiotemporal control of the synaptic terminal ATP production and Ca^2+^ concentration are essential for regulating the probability and rate of neurotransmitter release, quantal content, and kinetics of vesicle exocytosis and endocytosis [1,2,5]. For example, reciprocal cooperation between mitochondria and ER controls presynaptic Ca^2+^ handling and neurotransmitter release in lobster neuromuscular junction and bullfrog sympathetic ganglia [10,11], but not in the mouse calyx of Held [7], mouse neuromuscular junction [8] or goldfish mixed rod-cone bipolar (Mb1) cell [12]. Moreover, it is reported that mitochondria in the goldfish Mb1 cell terminals, which cluster far from the active zones [13], do not buffer presynaptic Ca^2+^ but instead primarily provide ATP to the PMCA to extrude intraterminal Ca^2+^ [12-14].

In contrast, vertebrate rod and cone photoreceptors are nonspiking neurons that maintain sustained depolarization and neurotransmitter release from ribbon synapses in darkness and produce light-dependent graded hyperpolarizing responses [15,16]. Following membrane depolarization, the kinetics of rapid exocytosis are faster in cones than rods, which is likely due to differences in presynaptic Ca^2+^ dynamics [17,18]. For example, salamander rods have one pool of vesicles that are released rapidly and linearly by submicromolar to micromolar concentrations of Ca^2+^ [17,19]: consistent with the single photon sensitivity and high fidelity of rods [20,21]. During darkness, photoreceptors maintain a sustained depolarization and continuously release glutamate [15,16,22,23]. To date, no comprehensive electrophysiological and pharmacological experiments examining the regulators of ATP production and Ca^2+^ homeostasis, similar to those conducted in several different neurons and goldfish retinal Mb1 cells [7,8,10-14,24], have been performed on rod or cone photoreceptor synaptic terminals. However, it has been reported that PMCA is the predominant Ca^2+^ extrusion mechanism in photoreceptor synaptic terminals [25] and that a ryanodine-dependent amplification mechanism is coupled to the release of glutamate in dark-adapted salamander rods [26,27]. In addition, studies with amphibian and mammalian retinas have demonstrated the presence of ER [28-31] and PMCA [25,32] in photoreceptor synaptic terminals.

Synaptic release of neurotransmitters at conventional synapses is rapid and transient [1,3,6-9]. The ATP-dependence of neurotransmitter uptake into synaptic vesicles, exocytotic vesicle priming and replenishment as well as the Ca^2+^-dependence of vesicle fusion and neurotransmitter exocytosis at synaptic membranes of neurons is widely accepted [1,3,4,33,34]. In contrast to conventional chemical synapses, the presynaptic terminals of retinal photoreceptors and bipolar cells possess ribbon synapses [35,36]. Ribbon synapses are specialized organelles located in nerve terminals that maintain a high rate of sustained transmitter release during a graded depolarization [35,36]. In addition, the photoreceptor and bipolar cell synaptic terminals differ from one another in their ribbon synaptic unit morphology, Ca^2+^ sensitivity for exocytosis, and kinetics of exocytosis and endocytosis [9,17,36,37]. Despite the kinetic differences of neurotransmitter release between conventional and ribbon synapses as well as among photoreceptor and bipolar cell ribbon synapses, studies suggest that mitochondrially-generated ATP is required for neurotransmitter uptake by synaptic vesicles, vesicle priming and vesicle competence, but not for vesicle fusion or neurotransmitter release [8,33,34,37,38].

Neuronal mitochondria play a fundamental role in regulating ATP production and intracellular Ca^2+^ for processes related to cellular metabolism, ionic homeostasis, neurotransmitter uptake/release and cell survival [1,2,39,40]. Energy demand drives mitochondrial ATP production [41]. ATP production is modulated by the total number of mitochondria per cell, size of the mitochondria, volume of the mitochondria per cell, and total surface area of the inner mitochondrial cristae membranes [42,43]. Neuronal somata are separated from their presynaptic terminals by relatively long axonal processes. This necessitates that mitochondria be differentially distributed within neurons to serve compartment-specific demands such as oxidative metabolism, neurotransmitter release, and Ca^2+^ homeostasis [2,6-8,44]. To develop a comprehensive knowledge and understanding of mammalian photoreceptor synaptic terminal ATP production, Ca^2+^ homeostasis and neurotransmitter kinetics, integrative immunocytochemical, ultrastructural and Ca^2+^ imaging studies on photoreceptor synaptic terminals, mitochondria and their interrelation with the proteins that utilize ATP and regulate Ca^2+^ concentrations are needed.

The retina has one of the highest rates of mitochondrial oxygen consumption and energy demand of any tissue [45]. The rod and cone photoreceptors contain at least 75% of the total retinal mitochondria, have the highest retinal cytochrome oxidase (COX) activity, and consume 2 fold to 3 fold more oxygen than the inner (proximal) retina [46-51]. To date, mitochondria have been localized to the photoreceptor inner segments (IS) and synaptic terminal regions [45-55]. Extrafoveal primate cone photoreceptor IS (CIS) have more mitochondria, higher COX activity, and stain more intensely for the Na^+^, K^+^-ATPase (NAKA) than rod photoreceptor IS (RIS) [46,47,51]. Murine CIS have two-fold more mitochondria than RIS and have higher COX activity than RIS [55]. In addition, CIS mitochondria have narrower crista junctions (CJs), greater cristae interconnectivity, and approximately 3 fold greater cristae membrane surface area than RIS mitochondria [55]. Furthermore, cones have significantly more synaptic ribbons in their terminals, possess unique basal junctions on their pedicles compared to rods, and have a higher concentration of ATP than rods [47,56-64]. Collectively, these biochemical, structural and substructural studies indicate that mammalian cones have an overall higher bioenergetic demand and ATP production than rods [55,56].

The overall goal of these studies was to develop a comprehensive structural and functional understanding of rod spherule and cone pedicle ribbon synaptic terminals. The specific aims of these laser scanning confocal microscopy (LSCM), electron microscopy (EM), three-dimensional electron tomography (ET), and Ca^2+^-fluo-3 imaging studies were four-fold. The first was to determine the cellular distribution and spatial interrelation of mitochondria, ER, PMCA, and NCX in the retina and especially in the photoreceptor synaptic terminals. The second was to determine if the cellular and subcellular ATP and Ca^2+^ regulatory mechanisms differed between rod spherule and cone pedicle synaptic terminals. The third was to determine if the relative [Ca^2+^] in rod spherules and cone pedicles differed during darkness and light-adaptation. The fourth goal was to determine and discuss the functional significance of our results in relation to rod and cone synaptic terminal bioenergetics, Ca^2+^ homeostasis and neurotransmitter release. Here we show that mitochondria, ER, PMCA and NCX each exhibited a distinct retinal lamination pattern. At higher spatial resolution, unique functionally related distributions of mitochondria, ER, PMCA and NCX were observed in both rod spherules and cone pedicles. Rod spherule and cone pedicle mitochondria also had marked functionally related differences in the location, total number, size, volume and total surface area of the inner mitochondrial cristae membranes. Furthermore, rod spherules maintained a lower [Ca^2+^] than cone pedicles during darkness, whereas cone pedicles lowered their intraterminal [Ca^2+^] faster and to a greater degree than rod spherules during light adaptation. These results provide a more comprehensive understanding of the spatiotemporal control of Ca^2+^ concentrations and mitochondrial ATP production in rod and cone ribbon synaptic terminals as they relate to exocytosis and endocytosis. Moreover, they provide important groundwork for further understanding compartmental differences in photoreceptors and offer new insight into strategies that will be necessary to treat visual deficits that result from rod and/or cone photoreceptor degeneration.

## Methods

### Materials

All chemicals were purchased as analytical or molecular biology grade from Sigma Chemical Co. (St. Louis, MO) or Fisher Scientific (Pittsburgh, PA) unless otherwise noted. The pH of all solutions was 7.40 at indicated temperatures.

### Experimental animals

All experimental and animal care procedures complied with the principles of the American Physiological Society, the NIH Guide for the Care and Use of Laboratory Animals and Maintenance (NIH publication No. 85-123, 1985) and were approved by the Institutional Animal Care and Use Committee of the University of Houston. Wild-type C57BL/6J mice (Harlan Sprague Dawley, Indianapolis, IN), from litters bred at our facility, were maintained on a 12:12 light:dark cycle (10-20 lux cage luminance) with food and water available ad libitum.

For most studies, 21 and 60 day old female mice were decapitated between one and two hours after light-onset and their eyes were rapidly removed and immersed in ice-cold phosphate buffered saline (PBS). For a few studies, 60 day old female mice were dark-adapted overnight and decapitated two hours after scheduled light-onset under dim red light (λ >650 nm). The corneas were gently punctured at the limbus. Then the eyes were either immersion-fixed in room temperature 4% paraformaldehyde in 0.1 M cacodylate buffer for 30 min for LSCM studies or in ice-cold 3% glutaraldehyde, 2% paraformaldehyde and 0.1% CaCl_2_ in 0.1 M cacodylate buffer (Karnovsky's fixative) for 12 h at 4 °C for conventional EM or ET studies as described [40,55,56,64]. Three to seven retinas from different mice were used for each independent analysis. There were no age-dependent differences on any analysis.

### Antibodies and lectins

The primary antibodies and lectin used in these studies were selected carefully for their tissue and cellular specificity, ability to recognize single or multiple protein isoforms, ability to recognize all splice variants of selected isoforms, and commercial availability (Table 1). We conducted extensive preliminary experiments to ensure that every antibody utilized in these studies had the appropriate specificity and penetration. All antibodies utilized in this study were titrated through a broad range of dilutions (most over 3 orders of magnitude) to determine optimal working dilutions. In addition, the concentration of Triton X-100 in the blocking agent was titrated to ensure optimal penetration without a significant loss in epitope. Immunolabeling specificity was confirmed by processing retinal sections as described below in the absence of the primary antibodies, by substituting normal rabbit or goat serum for polyclonal antibodies, or by using immunizing peptides for neutralization experiments. These procedures eliminated all specific labeling and revealed no false-positive labeling.

**Table 1:** Cell-specific primary antibodies and lectin.

**Primary antigen or lectin**	**Structure labeled**	**Host**	**Source**	**Dilution**	**References**
Calreticulin	ER	Rabbit	Chemicon	1:100	78,79
Middle wavelength-sensitive cone arrestin (M-CAr)	Cones	Rabbit	Kind gift from Cheryl Craft	1:1000	109
Cytochrome oxidase subunit IV (COX IV)	Mitochondrial inner membrane system	Mouse	Molecular Probes	1:500	40,73,94
Kinesin KIF3A	Photoreceptor ribbon and synaptic vesicles	Mouse	BD Biosciences	1:100	71
Na+/Ca2+ exchanger isoform 1 (NCX1)	Synaptic terminals	Rabbit	Swant	1:100	81,82
Middle wavelength-sensitive opsin (M-opsin)	Cones	Rabbit	Kind gift from Cheryl Craft	1:1000	65
Short wavelength-sensitive opsin (S-opsin)	Cones	Rabbit	Kind gift from Cheryl Craft	1:1000	65
pan-Plasma membrane Ca2+ ATPase (PMCA)	Synaptic terminals	Mouse	Affinity Bioreagents	1:100	81
Mitochondrial DNA polymerase-γ (POLG)	Mitochondrial matrix	Rabbit	Lab Vision	1:500	76
Protein kinase C-α (PKCα)	Rod bipolar cells	Rabbit	Sigma	1:1000	68,69
Rhodopsin (1D4)	Rods	Mouse	Chemicon	1:1000	72
Sarcoplasmic-endoplasmic reticulum Ca2+ ATPase isoform 3 (SERCA3)	ER	Rabbit	Affinity Bioreagents	1:400	80
Synaptotagmin 1	Photoreceptor and bipolar synaptic vesicles	Mouse	Chemicon	1:100	70,113
pan-Voltage-dependent anion channel (VDAC)	Mitochondrial outer membrane	Rabbit	Calbiochem	1:1000	74
Vesicular glutamate transporter 1 (VGluT1)	Photoreceptor and bipolar cell terminals	Guinea pig	Chemicon	1:1000	69
Peanut agglutinin (PNA)-Alexa Fluor 647 Conjugate	Cone outer segments and terminals		Molecular Probes	1:50	58,69

The primary antigen or lectin, structure(s) they label, host, source, and reference are presented.

An extensive panel of well-characterized primary antibodies directed against cell- and organelle-specific markers in the retina was used in double and triple labeling experiments. These were antibodies for rhodopsin, M-opsin, S-opsins, M-cone arrestin, vesicular glutamate transporter 1 (VGluT1), protein kinase C α (PKCα), kinesin KIF3A, synaptotagmin 1, and peanut agglutinin (PNA) [58,65-72]. The details about the other antibodies, previously not used for retinal immunocytochemistry studies, are immediately below. COX is the terminal electron transport complex of the mitochondrial respiratory chain and standard activity-dependent inner boundary membrane (IBM) and cristae marker [41,47]. The anti-COX subunit IV (COX IV) mouse monoclonal 20E8 antibody (Molecular Probes, Eugene, OR) is a molecular marker of the inner membrane system [73] and detects a single 16 kDa band on Western blots [40,105]. The anti-voltage-dependent anion channel (VDAC: mitochondrial porin) rabbit polyclonal antibody Ab-5 (Calbiochem, San Diego, CA) is an established marker of the outer mitochondrial membrane (OMM), was raised against amino acids 185-197 of the human VDAC, recognizes all three VDAC isoforms and detects a single 31 kDa band on Western blots [74]. We did not use the anti-VDAC mouse monoclonal antibody 31HL, used in retinal studies by Gincel et al. [104], since it only recognizes the VDAC1 isoform [75]. The anti-mitochondrial DNA polymerase-γ (POLG) rabbit polyclonal antibody Ab-1 (Lab Vision, Fremont, CA) is a nuclear-encoded protein responsible for mitochondrial DNA (mtDNA) repair and replication that is located in the mitochondrial matrix, was raised against amino acids 714-1061 of the human POLG, and detects a single 140 kDa band on Western blots [76]. POLG expression and message level are maintained regardless of the mtDNA status [77]. The anti-calreticulin rabbit polyclonal antibody AB3825 (Chemicon, Temecula, CA) is specific for the Ca^2+^-binding chaperone located in the lumen of all ER that actively modulates Ca^2+^ transport across the ER membrane [78]. It was raised against amino acids 412-417 of the C-terminus of calreticulin, does not cross react with other ER proteins and detects a single 60 kDa band on Western blots [79]. The anti-pan-sarcoplasmic-endoplasmic reticulum Ca^2+^ ATPase isoform 3 (SERCA3) rabbit polyclonal antibody PA-1-910A (Affinity BioReagents, Golden, CO) was raised against amino acids 29-39 of the mouse and rat SERCA3 isoform, recognizes all splice variants of human and rodent SERCA3, does not cross react with other SERCA isoforms, and detects a single 97 kDa band on Western blots [80]. The anti-pan-PMCA mouse monoclonal antibody MA3-914 (Affinity BioReagents) was raised against amino acids 724-783 of the human erythrocyte Ca^2+^ pump, recognizes all four isoforms of PMCA and detects a 140 kDa band on Western blots [81]. The anti-NCX1 rabbit polyclonal antibody p11-13 (Swant, Switzerland) was raised against the full length canine cardiac NCX1, recognizes all splice variants of NCX1, does not cross react with other NCX isoforms, and detects the 120 and 160 kDa bands on Western blots [81,82].

### Laser scanning confocal microscopy studies

Fixed eyes were rinsed in ice-cold PBS for 10 min. Fixed and washed eyes were cryoprotected in 30% sucrose/PBS solutions. The anterior segments were removed, eyecups were embedded in Tissue-Tek® OCT mounting media (Electron Microscopy Sciences, Fort Washington, PA) for 30 min and then frozen by immersion in liquid nitrogen. Retinas were sectioned along the vertical meridian on a cryostat at a thickness of 10-15 μm, collected onto Superfrost®/Plus microscope slides (Fisher Scientific) and stored at -20 °C until used.

For all LSCM experiments, sections were fixed and immunolabeled in parallel to insure identical processing. All analyzed sections were obtained 200-400 μm from the optic nerve head. Immunofluorescent labeling of frozen sections was essentially as described [79]. Briefly, sections were thawed for 60 min before use and postfixed by immersion in 4% paraformaldehyde for 15 min to improve tissue adherence to the slides. Sections were rinsed in nanopure water (npH_2_O), treated with 1% sodium borohydride to reduce nonspecific tissue autofluorescence, and immediately rinsed in npH_2_O. Sections were rinsed in PBS and treated for two hours at RT with 5% bovine serum albumin, 1% fish gelatin, 10% normal goat serum and 0.1-0.3% Triton X-100 in ice-cold PBS to block non-specific immunolabeling. Primary antibodies were applied for two days at 4 °C. For double and triple labeling experiments, primary antibodies from different host animals were applied simultaneously.

After incubation in primary antibody, the sections were rinsed three times in PBS and blocked for 30 min. Dilutions (1:500) of Cy3- or Cy5- (Jackson ImmunoResearch Laboratories, West Grove, PA) or Alexa Fluor 488 (Molecular Probes) -conjugated secondary antibodies were applied and incubated for 60 min in the dark at RT. For double and triple labeling experiments, secondary antibodies directed against primary antibodies from different species were applied simultaneously. After incubation with secondary antibody, the sections were rinsed in PBS and npH_2_O. The immunolabeled slides were dried and cover-slipped with Vectashield® anti-fade mounting medium (Vector Laboratories, Burlingame, CA) and stored at 4 °C until visualized. For double and triple labeling experiments using PNA, PNA-Alexa Fluor 647 (1:50 dilution: Molecular Probes) was applied simultaneously with the secondary antibody/antibodies.

LSCM images were acquired using a Leica TCS SP2 LSCM (Leica Microsystems, Exton, PA). Stacks of images from different Z-planes were obtained using a step size of 0.3-0.5 μm. "Bleedthrough" of fluorescent signals from different channels was eliminated by adjusting laser power, detector sensitivity and by sequentially imaging each fluorescent channel. Confocal images were identically and minimally processed by importing them into Adobe Photoshop CS software (Adobe Systems, Inc., Mountain View, CA). The results shown are representative of three to six separate immunolabeling experiments from three to five different mouse retinas. In all double and triple labeling experiments, the voxel dimensions in the X-Y dimensions were smaller than in the Z-dimension. Epitopes were designated as "colabeled" when the fluorescent pixels overlapped in the images. For all figures, the designation colabeled implies that the epitopes were within 290-400 nm of each other.

### Semi-quantitative assessment of immunolabeling intensity

The lamination-specific intensity of COX IV, VDAC, POLG, calreticulin, PMCA, and NCX immunolabeling was assessed by three independent viewers. Each viewer examined a minimum of five confocal immunofluorescent sections per retina from three to five mice and ranked the immunolabeling intensity on a relative five-point scale. The fluorescent labeling scale was intense (++++), strong (+++), moderate (++), weak (+) or absent (0). The combined results had a 90-95% concordance between viewers and are presented in Table 2.

**Table 2:** Retinal lamination and corresponding staining intensity of mitochondria, calcium transporters and ER.

	**Mitochondrial antibody**			**Calcium transporters**		
**Retinal Area or Structure**	**COX IV**	**VDAC**	**POLG**	**PMCA**	**NCX**	**Calreticulin**
ROS and COS	0	0	0	0	0	0
RIS and CIS	++++	++/+++	++++	+	++++	++++
Cone juxtanuclear mitochondria	+++/++++	+++/++++	+++	na	na	na
Rod juxtanuclear mitochondria	++/+++	++	+++	na	na	na
Overall ONL	+/++	++/+++	+/++	+	+++	++
OPL	++++	++++	++++	++++	++/+++	++/+++
Distal INL somas	++	+	++++	++	++	++/+++
Middle INL somas	+/++	+	+	++	++	++
Proximal INL somas	+/++	+	++++	++	++	++/+++
IPL sublamina-α	+++/++++	++/+++	+/++	+++	++/+++	0/+
IPL sublamina-β	+++/++++	++/+++	++	+++	++	0/+
RGC	+++	++	+++	++	++	+++
Müller glial end-feet	++++	0/+	+	++	0/+	0/+

COX IV represents cytochrome oxidase subunit IV; VDAC represents voltage-dependent anion channel; POLG represents mitochondrial DNA polymerase-γ; PMCA represents plasma membrane Ca^2+^ ATPase; NCX represents Na^+^/Ca^2+^ exchanger isoform 1; ER represents endoplasmic reticulum ROS and COS represents rod and cone outer segments; RIS and CIS represents rod and cone inner segments; ONL and INL represents outer and inner nuclear layer; OPL and IPL represents outer and inner plexiform layer; GCL represents ganglion cell layer intensity staining key: ++++ represents intense; +++ represents strong; ++ represents moderate; + represents weak; 0 represents absent; na represents not applicable.

### Conventional electron microscopy

The ultrastructure of mouse and rat photoreceptors has been described in several classic papers [28,53,83,84]. The fixation procedures used in these studies preserved the ultrastructure of the outer retina, although they were not optimal for maintaining the photoreceptor mitochondria ultrastructure and substructure as these were not the major goals of these studies. In contrast, one of our primary goals was to analyze and compare the ultrastructural and substructural features and characteristics of rod and cone photoreceptor mitochondria. Therefore, we used our well-validated fixation and embedding procedures for these endeavors, essentially as described [40,55,65,85]. Briefly, each eye was fixed overnight and a piece of the superior temporal retina 200-250 μm from the optic nerve was obtained. We chose this retinal area for two reasons. First, we used the same area of mouse retina for our previous ultrastructural and ET work on mouse cone inner segment mitochondria [55]. This allowed us to compare directly our results from rod and cone inner segment mitochondria to the current study on rod and cone synaptic terminal mitochondria. Second, this region contains mostly middle wavelength-sensitive (M) cones [169] and M cones in the mouse are similar to those in other mammals [86,87], which enables cross-species comparisons. Sections were dehydrated and embedded in Spurr's or Araldite resin as described [40,55,65,88]. Ultra-thin vertical sections of the retina were stained with uranyl acetate and lead citrate before being examined in a JEOL 100-C or 1200EX transmission EM (Tokyo, Japan). The number of mitochondria per rod spherule and cone pedicle was calculated from three to five different grids from each of five different mice. The mean number from each mouse was determined and the overall mean±SEM was calculated.

### Three-dimensional electron microscope tomography

Mouse retinas were prepared for ET essentially as described [40,55]. Briefly, the superior temporal retina 200-250 μm from the optic nerve was trimmed (vide supra) and the retinal sections were dehydrated, embedded in Durcupan resin, sectioned (500 nm thick) and imaged using the single- and double-tilt series techniques described by Perkins and co-workers [55,89,90]. Fiducial cues, consisting of 20 nm colloidal gold particles, were deposited on both sides of the section. For each reconstruction, a series of images at regular tilt increments was collected with a JEOL 4000EX intermediate-voltage EM operated at 400 kV. To limit anisotropic specimen thinning during image collection, the specimens were irradiated before each tilt series. Tilt series were recorded at 20,000X magnification with an angular increment of 2 ° from -60 ° to +60 ° about an axis perpendicular to the optical axis of the microscope. A computer-controlled goniometer accurately incremented the angular steps. A slow-scan CCD camera with pixel dimensions of 1960x2560 was used to collect images. The pixel resolution was 1.1 nm. Illumination was held to near parallel beam conditions and constant optical density was maintained constant by varying the exposure time. The IMOD package [91] was used for rough alignment with the fine alignment and reconstruction performed using the TxBR package [92].

Volume segmentation was performed by manual tracing in the planes of highest resolution with the program Xvoxtrace [90]. The mitochondrial reconstructions were visualized using Analyze (Mayo Foundation, Rochester, MN) or the surface-rendering graphics of Synu (National Center for Microscopy and Imaging Research, San Diego, CA) as previously described [55,93]. These programs allow one to step through slices of the reconstruction in any orientation and to track or model features of interest in three dimensions. Measurements of structural features were made within segmented volumes by the programs Synuarea and Synuvolume (National Center for Microscopy and Imaging Research, San Diego, CA). Overall, measurements from tomographic reconstructions were made from seven distinct mitochondria (four from rods and three from cones) using retinas obtained from three different mice.

### Ca^2+^ imaging and correlative electron microscopy of rod and cone synaptic terminals in dark-adapted and light-adapted whole retinas

Our fluo-3 Ca^2+^ imaging and LSCM procedures [40,94], with modifications as described, were used to localize the distribution and to determine the relative concentrations of free Ca^2+^ in dark- and light-adapted rod and cone synaptic terminals. All dark-adapted procedures were conducted under dim red light (λ >650 nm). Whole neural retinas were isolated from dark-adapted mice (n=3 mice), incubated in a Ca^2+^-free HEPES buffer (30 mM HEPES, 125 mM NaCl, 5 mM KCl, 3 mM MgCl_2_, 10 mM D-glucose: pH 7.4, 310±3 mOsm) containing rhodamine-labeled PNA (1:10 dilution; Vector Laboratories, Burlingame, CA) and bovine serum albumin (1 mg/ml) for 10 min at RT followed by three gentle aspiration/rinses with Ca^2+^-free HEPES buffer. Then the retinas were incubated in the Ca^2+^-free HEPES buffer containing 3 mM fluo-3 AM and 0.025% pluronic acid (Molecular Probes) for 30 min at RT followed by a gentle aspiration/rinse in Ca^2+^-free HEPES buffer. Preliminary experiments determined that the organic anion transport inhibitor probenecid (2.5 mM) did not significantly affect the fluo-3 fluorescence results in the rod or cone synaptic terminals, so it was not used in the present experiments. We did not use verapamil, an inhibitor of the multidrug resistance pump, because it also blocks L-type Ca^2+^ channels and would have confounded our results [40,94].

The retinas were mounted retinal ganglion cell side down on nitrocellulose filter paper and several 100-150 μm thick slices were made in the central retinal area essentially as described [95]. The retinal slices and filter paper were placed on small volume glass bottom dishes coated with Matrigel (Collaborative Research, Palo Alto, CA) and incubated for 15 min at 27 °C in HEPES buffer containing 1.5 mM CaCl_2_ in order to restore the normal extracellular Ca^2+^ concentration and increase the esterase activity [96]. The HEPES buffer with CaCl_2_ was aspirated and replaced with fresh buffer prior to the onset of Ca^2+^ imaging.

To confirm that the observed fluorescence signals reflected changes in internal Ca^2+^ levels, we conducted three different experiments. First, we added 1 mM Pb^2+^, a potent fluo-3 fluorescence quencher [Kd for fluo-3 is 6 pM: 94,97], to the Ca^2+^-containing HEPES buffer. Similar to our previous results [40,94], this significantly quenched the Ca^2+^-enhanced fluo-3 fluorescence in dark-adapted photoreceptor synaptic terminals (data not shown). Second, we incubated retinal slices in Ca^2+^-free HEPES buffer with 5 mM BAPTA-AM [1,2-bis(o-aminophenoxy)ethane-N,N,N',N'-tetraacetic acid] for 15 min at 27 °C. The fluo-3 fluorescence measured in dark-adapted photoreceptor synaptic terminals, from these retinal slices, was not above background (data not shown). Third, we attempted an in vivo calibration of fluo-3 fluorescence in photoreceptor synaptic terminals using the standard ionomycin, Mn^2+^ and digitonin procedure for NIH 3T3 cells [98,99] and isolated cerebellar granule cells [97] as well as the Ca^2+^ ionophore A-23187. These calibration procedures saturated the fluo-3 fluorescent signal above the physiological range. However, they were unreliable because shortly after the fluo-3 fluorescent signal saturated the rod photoreceptors initiated apoptosis: as we reported [40,94]. Therefore, we established an in vitro relative fluorescence intensity (RFI) standard curve, as described [98,100], in order to estimate the free [Ca^2+^] in the photoreceptor synaptic terminals. An intracellular buffer (25 mM HEPES, 130 mM KCl, 5 mM NaCl, 3 mM MgCl_2_, pH 7.2, 305±3 mOsm) that contained calibrated Ca^2+^ buffers (0-40 μM Ca^2+^; Molecular Probes) and 5 μM of the penta-ammonium salt of fluo-3 (Molecular Probes) was pipetted onto dual concave glass slides maintained in the dark at 27 °C. Fluo-3 fluorescence was measured with the Zeiss LSM-410 confocal microscope system (Zeiss, Thornwood, NY) as described below.

Images were acquired on a Zeiss LSM-410 confocal microscope utilizing a Zeiss Axiovert 100 microscope equipped with an X63 oil immersion objective (1.4 numerical aperature). An argon laser excited fluo-3 at 488 nm and PNA-rhodamine at 568 nm, and bandpass filters of 530 and 590 nm collected the signals from fluo-3 and rhodamine, respectively. For each retina and all calibration procedures, the gain on the confocal system was kept constant for all recording conditions. To minimize light exposure to the retina and photobleaching, the OPL was identified rapidly and at low confocal gain by its PNA fluorescence and its retinal location. Once identified, the retinas were dark-adapted for an additional five minutes. Ten to 15 optical sections of the dark-adapted OPL were obtained using a Z-axis step size of 0.5 μm. Following these recordings, the retinas were dark-adapted for five minutes and then a rod saturating light illuminated the retina [49,97]. Ca^2+^ image recordings began one minute after light onset as the light-adapted decrease in photoreceptor oxygen consumption stabilizes during the first minute of the light stimulation [49,50,101].

Z-axis reconstructions were made using Zeiss software. The image was a Z-stack maximum projection (single confocal section). For these experiments, the XY resolution was 200-250 nm and the Z resolution was 300 nm. The RFI of Ca^2+^-fluo-3 in dark- and light-adapted rod spherules and cone pedicles was determined using NIH Image, version 1.62. To directly compare the RFI, and thus the relative free [Ca^2+^], in dark- and light-adapted rod and cone synaptic terminals, only experiments where relatively adjacent photoreceptor synaptic terminals were imaged are included in this data set. To normalize all the images a standard background subtraction was performed on each image. Then the overall RFI within each dark- and light-adapted rod spherule and cone pedicle was determined based on a grey scale with 256 levels. The light-adapted to dark-adapted fluorescence intensity ratio for each rod spherule and cone pedicle from each retina was calculated, means±SEMs were determined, and the data was statistically analyzed. The mean RFI of dark-adapted rod spherules and cone pedicles as well as light-adapted rod spherules and cone pedicles were determined and compared. To analyze and evaluate this data, the RFI values were normalized by the gain of the confocal system expressed on a linear scale as described [102]. The normalized intensity values were calculated, means±SEMs were determined, and the data was statistically analyzed. Similar imaging procedures were used to establish the Ca^2+^ and fluo-3 fluorescence calibration curve.

Confocal images for each retina were identically and minimally processed by importing them into Adobe Photoshop CS software (Adobe Systems, Inc., Mountain View, CA). For higher resolution and better visualization of the Ca^2+^ microdomains, the pseudocolored images were transformed using the advanced "stained glass" imaging synthesis algorithm provided in Adobe Photoshop under the Filter and Texture pull-down menus (Adobe Systems, Inc.). Although the exact algorithm is proprietary, the values for cell size, border thickness and light intensity were 10, 1, and 2, respectively. The results shown are from a representative retina from three separate experiments from three different mouse retinas.

Conventional EM identified the rod spherules and cone pedicles from where the Ca^2+^ images were obtained. After the experiment, the small glass dish was placed on ice and the retinal slice was fixed for 30 min with ice-cold Karnovsky's fixative (described above). Then the dish and retina were placed in a larger volume of fresh Karnovsky's fixative for 12 h at 4 °C and the tissue was processed for conventional EM as described.

### Statistical analysis

The electron tomography and fluo-3 intensity data were analyzed using a two-tailed Student's t-test (Kaleidagraph Synergy Software, Reading, PA). The differences were considered significant if p<0.05. Data are presented as means±SEM.

## Results

### Topographically distinct mitochondrial antibodies reveal differential lamination and cellular distribution of retinal mitochondria: LSCM and EM studies

**Figure 1:**
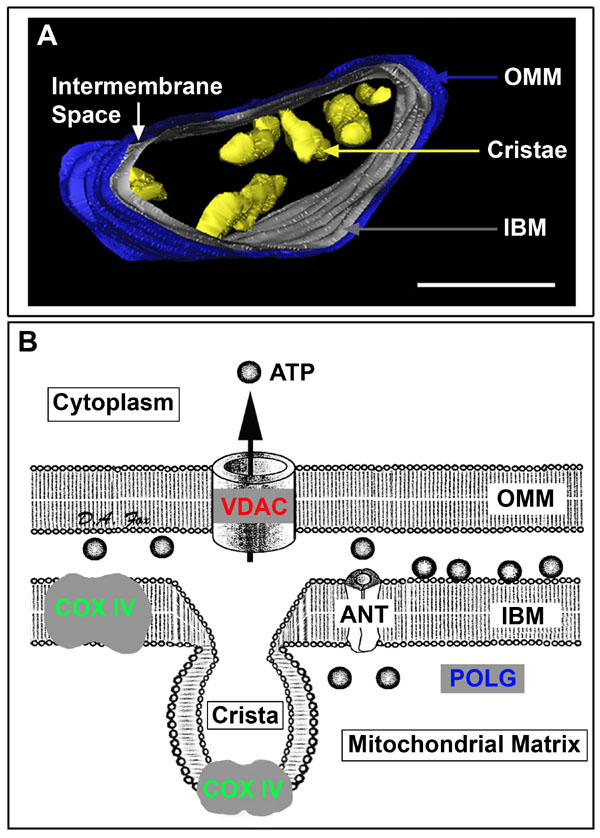
Rod inner segment mitochondrion and topographical markers of mitochondrial compartments. **A**: View of a mouse rod inner segment mitochondrion from a three-dimensional electron tomographic study after volume segmentation. The outer mitochondrial membrane (OMM) is blue, inner boundary membrane (IBM) apposed to the OMM is grey, cristae are yellow and a white arrow identifies the intermembrane space between the OMM and IBM. The IBM and cristal membranes form the contiguous, but distinct inner membrane system of the mitochondria [84,89,124,128]. Most cristae were removed graphically for illustrative purposes (adapted from [4]). Scale bar equals 200 nm. **B**: Schematic drawing of mitochondrial topology with site-specific compartments targeted for immunocytochemical experiments. Common immunocytochemical markers of the OMM (voltage-dependent anion channel: VDAC), inner membrane system (cytochrome oxidase IV: COX IV), and mitochondrial matrix (mitochondrial DNA polymerase-γ: POLG) are depicted. These mitochondrial compartments are consistent with the rod mitochondrion illustrated in **A** and the single label colorized confocal images shown in Figure 2A,B,D. Note the proximity of COX IV and VDAC to the adenine nucleotide transporter (ANT). The abbreviations COX IV, VDAC, and POLG are used in all subsequent figures.

**Figure 2:**
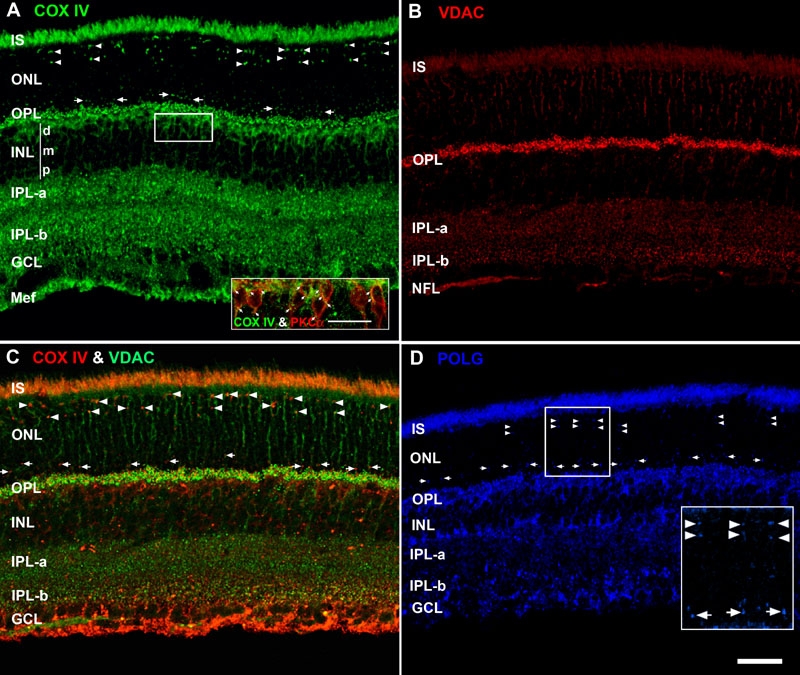
Molecular markers for three separate mitochondrial compartments reveal distinct retinal distribution and lamination patterns. The abbreviations of the retinal layers are used for this and all subsequent figures. IS represents inner segments, ONL represents outer nuclear layer, OPL represents outer plexiform layer, INL represents inner nuclear layer (d: distal, m: middle, p: proximal), IPL-α represents inner plexiform layer sublamina-α (OFF lamina), IPL-β: inner plexiform layer sublamina-β (ON lamina), GCL: ganglion cell layer, and Mef: Müller glial end-feet. **A**: Confocal image of retina immunolabeled for COX IV. The pairs of white arrowheads identify numerous intensely labeled arc-shaped puncta located in the distal ONL, which are juxtanuclear mitochondria in the cone somas. Between the white arrows, in the proximal ONL, is a band of small circular puncta that are juxtanuclear mitochondria near rod nuclei. The inset shows colocalization of COX IV (green) and PKCα (red) in rod bipolar cells (white arrows): scale bar equal 20 μm. **B**: Confocal image of retina immunostained for VDAC. Note the intense labeling in the OPL. **C**: Confocal image of retina double labeled with antibodies against COX IV (red) and VDAC (green). The pseudocoloring in panels A and B was reversed in this panel to clearly show the colocalization (yellow-orange pixels) in the IS, cone juxtanuclear mitochondria (white arrowheads) and rod juxtanuclear mitochondria (white arrows). Punctate colocalization also is present throughout the OPL, IPL-α, and IPL-β. **D**: Confocal image of retina immunolabeled for POLG. POLG also labels the cone juxtanuclear mitochondria (white arrowheads) and rod juxtanuclear mitochondria (white arrows). The inset is a higher magnification (2X) view. Scale bar equal 40 μm for all panels.

**Figure 3:**
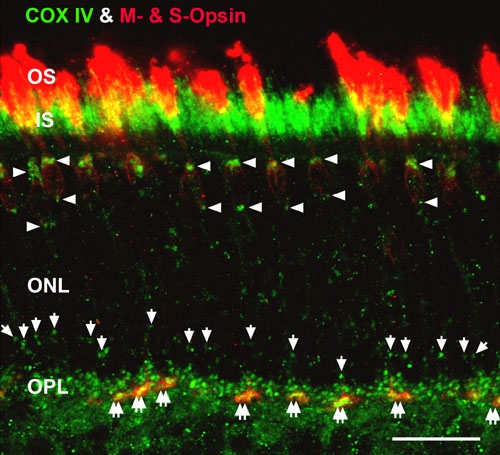
High magnification confocal image of cone juxtanuclear mitochondria and clusters of mitochondria in cone pedicles. Retinas were double labeled for COX IV (green) and the M- and S-cone opsins (red). The yellow-orange pixels show that COX IV and the cone opsins were in close apposition in the CIS and cone pedicles (double white arrows). Numerous large COX IV-positive juxtanuclear mitochondria are located above and below the distal cone nuclei (white arrowheads), while smaller COX IV-positive juxtanuclear mitochondria are located above rod nuclei (white arrows). Scale bar equal 20 μm.

**Figure 4:**
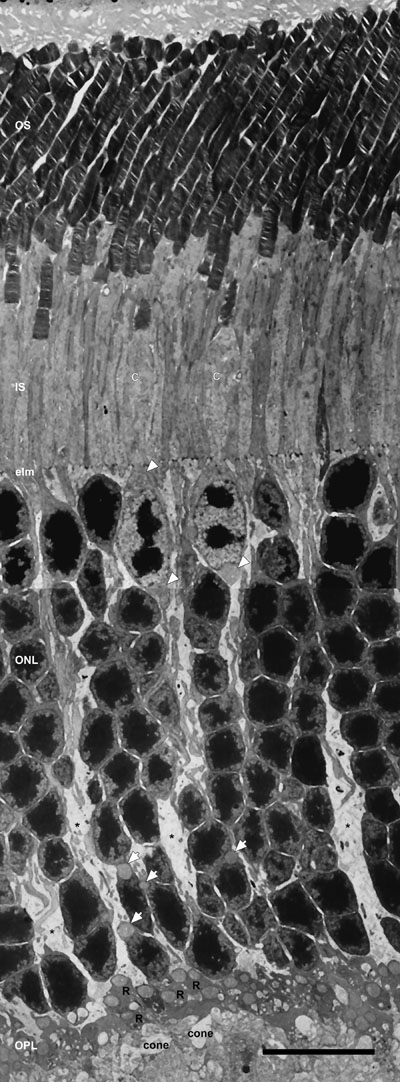
Low-magnification electron micrograph of longitudinal section of the entire photoreceptor layer. Rod and cone (C) photoreceptors are distinguished by several morphological differences. Rod inner segments (IS) are longer, thicker and located more distally in the retina, whereas cone IS are larger and more electron lucent. The cone nuclei are located in the outer third of the outer nuclear layer (ONL), contain several clumps of irregularly shaped heterochromatin and possess two juxtanuclear mitochondria: one above and one below the nucleus (black outlined white arrowheads). The large mitochondrion at the base of the cone nuclei is located at the origin of the wide cone axon, as previously noted [53]. Rod nuclei are present throughout the ONL and contain a single compact mass of heterochromatin. Single juxtanuclear mitochondria are present in many rod somas located in the inner third of the ONL (black outlined white arrows). Numerous synaptic terminals, with large mitochondria, are in the outer plexiform (OPL). Three to five tiers of dark-staining rod spherules (R) overly a single row of more electron lucent cone pedicles (cone) that contain multiple mitochondria. Müller glial cell processes extend throughout the OPL and ONL, and terminate at the external limiting membrane (elm). Scale bar equal 10 μm.

Photoreceptors contain about 75% of the retinal mitochondria, have the highest COX activity, and have significantly greater oxygen consumption than inner retina [46-51]. To test the hypothesis that the distribution of retinal mitochondria reflects cell-specific and synaptic-specific differences in bioenergetics, we examined the immunofluorescent staining pattern of three independent topographical markers of mitochondrial membranes and compartments: COX IV, VDAC, and POLG (Figure 1; Table 1 and Table 2). Figure 1 illustrates the three topographically distinct mitochondrial regions that were recognized by the selected antibodies: the OMM, inner membrane system that includes the IBM and cristal membranes, and mitochondrial matrix. Below, we describe the typical labeling patterns, from outer or distal retina to inner or proximal retina, for each of these three markers. No specific labeling of the rod or cone outer segments (ROS; COS) was observed with any mitochondrial antibody, consistent with the absence of mitochondria in this photoreceptor compartment [53,65]. Intense COX IV immunolabeling was present in both RIS and CIS mitochondria (Figure 2A and Figure 3) consistent with our previous results and the concentration of mitochondria in the IS [55]. At first glance, the outer nuclear layer (ONL) contained relatively few mitochondria. However, a more detailed analysis of the ONL revealed two distinct COX IV-positive mitochondrial populations in the ONL. The first population was located in the distal ONL where strong to intense COX IV-positive arc-shaped puncta were detected (Figure 2A: white arrowheads). Double labeling experiments, with a mixture of middle and short wavelength-sensitive cone opsin antibodies (Table 1), revealed that these previously undescribed pair of juxtanuclear mitochondria were located above and below the most distal cone nuclei (Figure 3: pairs of white arrowheads). Electron microscopy confirmed this result (Figure 4: white and black arrowheads). Double labeling experiments with COX IV and VDAC revealed that these two mitochondrial markers colocalized in the juxtanuclear mitochondria (Figure 2C: white arrowheads; yellow-orange pixels). The second mitochondrial population in the ONL was a band of smaller, moderately labeled puncta located in the proximal ONL (Figure 2A,D and Figure 3: white arrows). These puncta are juxtanuclear mitochondria localized next to individual rod nuclei present in the proximal ONL, as confirmed by electron microscopy (Figure 4: white and black arrows). Moreover, COX IV and VDAC colocalized throughout the OPL (Figure 2C).

The IS and outer plexiform layer (OPL) intensely labeled for COX IV (Figure 2A and Figure 3), confirming that mammalian photoreceptor IS and synaptic terminals contain numerous highly active mitochondria [47,55,62]. A mixture of M- and S-cone opsin antibodies [65] labeled all the cones in the mouse retina [66]. In addition, the high magnification confocal image of COX IV and cone opsin double labeling revealed a laminar COX IV staining pattern in the OPL (Figure 3). That is, a single row of large opsin-positive cone pedicles was present in the proximal OPL that contained numerous COX IV-positive puncta (Figure 3: double white arrows). In contrast, the distal OPL had several rows of opsin-negative terminals that contained large round COX IV-positive spheres (Figure 3). These observations suggest that rod spherules contain a single large mitochondrion, whereas cone pedicles contain numerous, albeit smaller, mitochondria. To directly investigate this we conducted EM studies as described below. In summary, the overall staining pattern and intensity of COX IV immunofluorescence in photoreceptors indicates that most of their aerobically-generated ATP is produced by IS and synaptic terminal mitochondria (Table 2).

The proximal (inner) retina also exhibited a differential distribution of COX IV immunolabeling. The inner nuclear layer (INL) somas had weak to moderate COX IV labeling with the strongest staining located in the distal INL (Figure 2A). The location of these distal somas and their Chx10-positive staining (data not shown; manuscript in preparation) indicates that they are bipolar cells [67,68,103]. A subpopulation of these Chx10-positive bipolar cell somas were COX IV- and PKCα-positive (Figure 2A: inset), confirming that they are rod bipolar cells [67,68]. Less intense staining was detectable in the middle and proximal INL somas: the size and location of these somas suggested they were Müller glial somas and amacrine cells, respectively [68]. COX IV strongly to intensely labeled the inner plexiform layer (IPL), ganglion cell layer (GCL) and distal Müller glial end-feet. A clear demarcation between the OFF (sublamina-α: IPL-α) and ON (sublamina-β: IPL-β) IPL sublamina was observed (Figure 2A).

**Figure 5:**
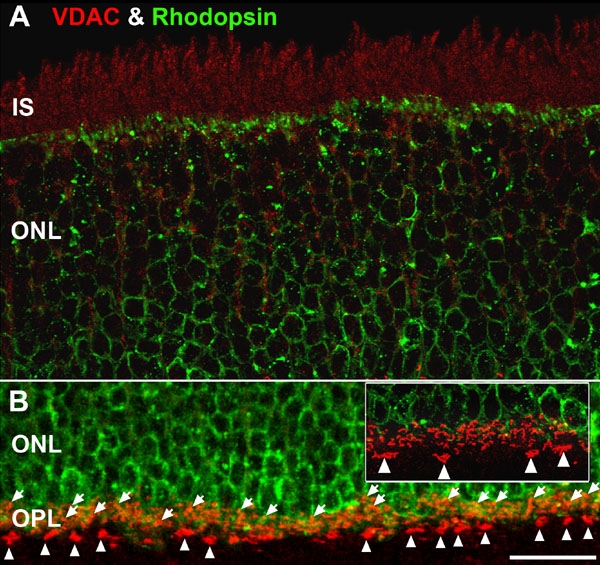
High magnification confocal images of outer retina double labeled for VDAC (red) and rhodopsin (green). **A**: To reveal the voltage-dependent anion channel (VDAC) labeling of the IS (Figure 2B) the prominent rhodopsin labeling in the OSs and most IS was removed using Photoshop. Minimal colocalization of VDAC and rhodopsin occur in the ONL, highlighting the lack of mitochondria in most rod somas. However, the Müller glial cell processes that surround the rod somas are VDAC positive (data not shown). **B**: Stratification of rod spherule and cone pedicle mitochondria in the OPL. Double labeling for VDAC and rhodopsin shows the distal-proximal stratification of rod spherules (white arrows) and cone pedicles and their associated mitochondria. Tiers of rod spherules, each with one large mitochondrion, overly the rhodopsin-positive region. The more proximal cone pedicles contain clusters of mitochondria (white arrowheads) and are located in the rhodopsin-negative region. The inset is a higher magnification (2X) view of the OPL. Scale bar equal 20 μm for both panels.

Figure 2B,C show that moderate to strong VDAC labeling was present throughout the IS and that VDAC and COX IV colocalized in this compartment. Thin VDAC-positive processes are evident throughout the ONL. These VDAC-positive processes surrounded the tightly packed rhodopsin-positive somas in the ONL (Figure 5), suggesting that they are Müller glial cell processes. EM studies confirmed this (Figure 4). Cone and rod juxtanuclear mitochondria strongly colabeled for VDAC and COX IV (Figure 2C). The most intense VDAC labeling occurred throughout the OPL and this colocalized with COX IV (Figure 2C). In contrast to the ONL, the INL weakly stained for VDAC. Moderate to strong VDAC labeling was evident throughout the IPL. However, stronger punctate labeling occurred in the most proximal band of IPL-β, where the rod bipolar cells terminate [67,68]. Double labeling experiments showed that VDAC and COX IV colocalized in these rod bipolar cell terminals (Figure 2C). Triple labeling experiments with VDAC, PKCα, and VGluT1 confirmed that these were rod bipolar cell terminals (data not shown; manuscript in preparation). The GCL and proximal Müller glial cell end-feet exhibited weak VDAC labeling.

Figure 2D shows that POLG intensely labeled the IS, OPL, distal and proximal INL somas, proximal IPL-β, and GCL. Similar to the COX IV staining pattern in the ONL, there were strong POLG-positive arc-shaped puncta in the distal ONL (white arrowheads) and circular puncta in the proximal ONL (white arrows). Except for the presumed rod bipolar cell terminals in IPL-β, the remainder of the IPL exhibited relatively moderate staining. The COX IV, VDAC, and POLG labeling patterns were similar in sections obtained from the superior and inferior central retina.

### Endoplasmic reticulum are differentially distributed throughout the retina

**Figure 6:**
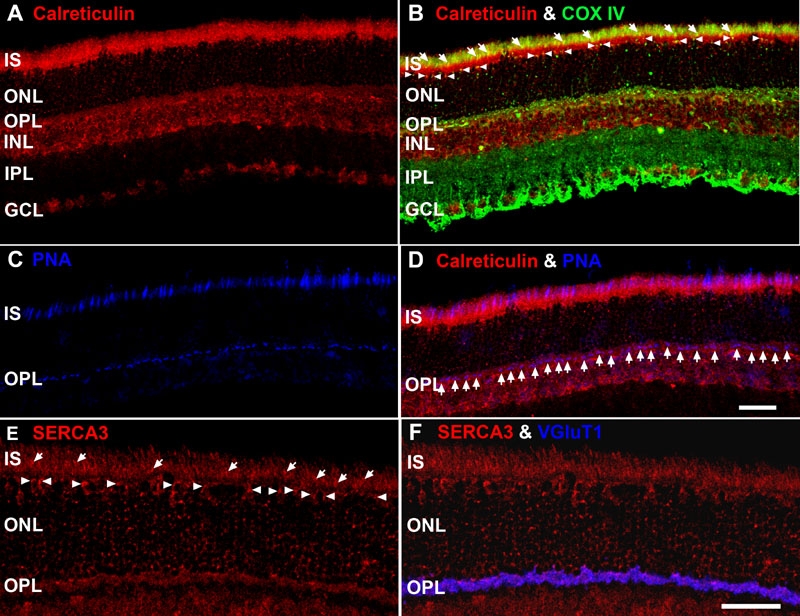
Molecular markers of endoplasmic reticulum (ER) reveal distinct retinal distribution and lamination patterns. **A**-**D**: Confocal image of retina triple labeled for calreticulin, COX IV and PNA. Scale bar equal 20 μm. **A**: Retina stained for calreticulin, a Ca^2+^-binding protein present in all ER lumen. Note the intense labeling in the IS, OPL and INL. **B**: Location of calreticulin (red) and COX IV (green). These proteins are in close apposition in the ellipsoid region of photoreceptor IS (white arrows and yellow pixels), juxtanuclear mitochondria associated with cone somas (white arrowheads) and in the OPL (punctate yellow-orange pixels). **C**: Retina stained with peanut agglutinin (PNA), a relatively selective marker for cones, shows distinct labeling in the IS and OPL. **D**: Calreticulin (red) and PNA (blue) colocalize in CIS and near the pedicles (white arrows; bright purple pixels). **E**: Pan-Smooth ER Ca^2+^ ATPase isoform 3 (SERCA3) immunolabeling. This antibody labels in close apposition to the RIS and CIS (white arrows), juxtanuclear mitochondria associated with cone somas (white arrowheads) and the mitochondria in the OPL. **F**: Colocalization of pan-SERCA3 (red) and VGluT1 (blue) throughout the OPL, indicating the presence of ER in the photoreceptor synaptic terminals. Scale bar for panels **E** and **F** equal 40 μm.

Several LSCM, EM, and ET studies have shown that ER and mitochondria are in close apposition and that microdomains of high Ca^2+^ are shared between these organelles [2,37,89,93,104,105]. To directly test the hypothesis that ER are closely apposed to retinal mitochondria, and especially those in the photoreceptor synaptic terminals, we examined the staining pattern of two independent ER markers in combination with COX IV or VGluT1 (Figure 6; Table 1 and Table 2) and conducted EM studies (vide infra). Figure 6A shows that calreticulin, a molecular marker of all ER [75], intensely labeled the entire IS region and that the OPL, INL, and GCL were moderately to strongly labeled. In contrast, the ONL and IPL weakly labeled for calreticulin. Calreticulin and COX IV colocalized in multiple retinal areas (Figure 6B: punctate yellowish pixels), indicating that these two proteins were in close apposition. This interrelation was especially prominent throughout the photoreceptor ellipsoid region. Moreover, calreticulin was in close apposition to the cone juxtanuclear mitochondria (white arrowheads) and mitochondria in the OPL. Labeling in the latter revealed that calreticulin was located close to COX IV-positive synaptic terminal mitochondria (see OPL in Figure 3). Calreticulin and COX IV also colocalized in the INL as evidenced by the diffuse orange-colored pixels. Figure 6C,D further illustrate that calreticulin is expressed intensely in CIS and cone pedicles (Figure 6D: white arrows and purple pixels).

To determine if another ER antibody colocalized in photoreceptor synaptic terminals, single and double label experiments were conducted with the SERCA3 and VGluT1 antibodies. We did not utilize the SERCA N89 polyclonal rabbit antibody as it labels COS and photoreceptor IS, but only sparsely labels photoreceptor synaptic terminals in adult mouse, rat and monkey [31]. Figure 6E shows that the SERCA3 moderately to strongly labeled ER in the RIS and CIS (white arrows), closely apposed to the cone juxtanuclear mitochondria (white arrowheads), and in the OPL. Thus, the SERCA3 labeling pattern in the outer (distal) retina was similar to that of calreticulin. Double labeling experiments with the pan-SERCA3 and VGluT1 revealed that they completely and strongly colocalized in both rod spherules and cone pedicles (Figure 6F: purple pixels).

### PMCA and NCX1 differentially and selectively label the rod spherules and cone pedicles, respectively

**Figure 7:**
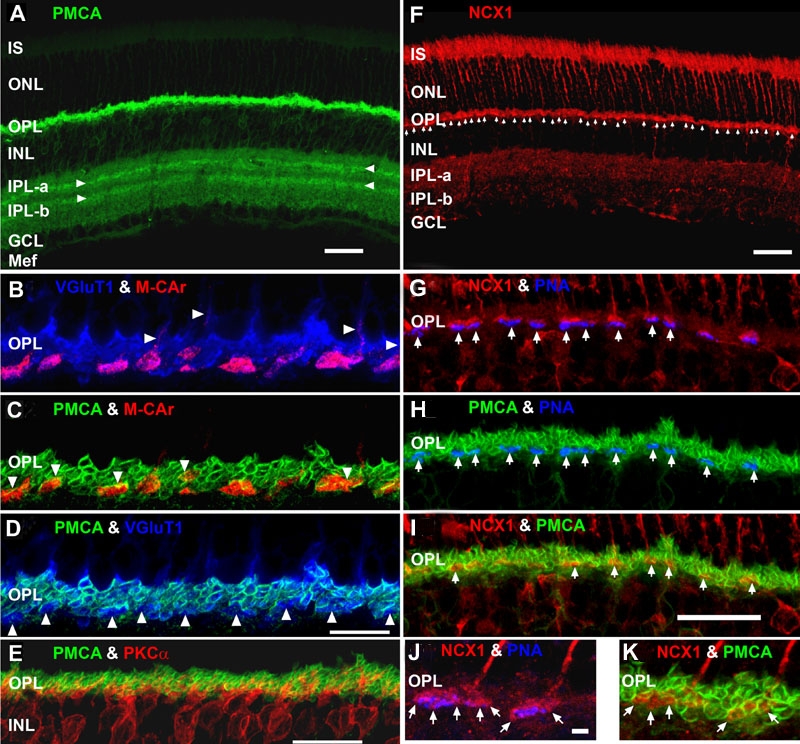
Lamination patterns and differential compartmentation of pan-plasma membrane Ca^2+^ ATPase and Na^+^-Ca^2+^ exchanger isoform 1 in rod spherules and cone pedicles. **A**: Confocal image of retina immunolabeled for pan-plasma membrane Ca^2+^ ATPase (pan-PMCA). The double white arrowheads identify strongly labeled PMCA bands in each IPL sublamina. The scale bar applies to **A**, **D** and represents 40 μm. **B**-**D**: PMCA preferentially labels rod spherules. OPL double labeled with markers for PMCA (green), vesicular glutamate transporter 1 (VGluT1: blue) and/or M-cone arrestin (M-CAr: red). Scale bar equal 20 μm. **B**: VGluT1, which labels photoreceptor terminals and M-CAr, which labels cones, colocalize in the OPL. This reveals the large dome-shaped cone pedicles (purple pixels) and some cone axons (white arrowheads). **C**: This high magnification image shows the horseshoe-like appearance of the PMCA-positive rod spherules. In contrast, the retina double labeled with PMCA and M-CAr shows that cone pedicles stain weakly and diffusely for PMCA, except for a discrete band at the top of the pedicle (white arrowheads: yellow pixels). **D**: A retina double labeled with PMCA and VGluT1 confirms that PMCA extensively labels rod spherules, but only sparsely labels cone pedicles (white arrowheads). **E**: PKCα-positive rod bipolar cells (red) are pan-PMCA-negative (green), although they are in close apposition around the rod spherules (yellow pixels). The scale bar represents 20 μm. **F**: Retinal localization of Na^+^-Ca^2+^ exchanger isoform 1 (NCX1). NCX1 intensely labels IS, cone axons and cone pedicles (white arrowheads). **G**-**I**: NCX1 preferentially labels cone pedicles. OPL from triple labeled experiments with markers for NCX1 (red), PMCA (green), and/or PNA (blue). Scale bar equal 20 μm. **G**: PNA stains selected flat contact regions on the large cone terminal (white arrows). NCX1 intensely labeled the entire cone pedicle and axon terminals, whereas rod spherules were diffusely labeled. **H**: PMCA labels the rod spherule membranes with a horseshoe-like appearance, but does not prominently colocalize with PNA-positive cone pedicle membranes (white arrows). **I**: Retina double labeled with PMCA and NCX1 shows that these proteins colocalized in cone pedicles near the axon junction (white arrows: yellow pixels). **J**, **K**: High magnification confocal images showing preferential labeling of cone pedicles by NCX1 and rod spherules by PMCA. OPL from triple labeled experiments with markers for NCX1 (red), PMCA (green), and/or PNA (blue). Scale bar equals to 10 μm. **J**: Double labeled retina shows that NCX1 intensely labels the entire cone pedicle and axon terminals, but only diffusely labels the rod spherules. This high magnification image also shows that PNA stains selected flat contact regions on the large cone terminal, whereas invaginating synaptic regions (white arrows) are not labeled by PNA. **K**: Double labeled retina shows that PMCA and NCX1 colocalize in cone pedicles near the axon junction (white arrows: yellow pixels).

PMCA and NCX share a complementary role in regulating the presynaptic Ca^2+^ concentration. PMCA has a high affinity and low turnover rate, whereas NCX has a low affinity and high turnover rate for Ca^2+^ extrusion [75,76]. Few studies have examined the location or functional roles of the two major presynaptic Ca^2+^ transporters in neurons [6,25,106-108]. The kinetics of exocytosis is 10 fold faster in cones than in rods [17,18], suggesting that PMCA and NCX may be differentially distributed or located in rod and cone synaptic terminals. To directly test this hypothesis, the staining pattern of PMCA and NCX1 in combination with markers of photoreceptor cell terminals [69] or cones [57,58] was examined (Figure 7; Table 1 and Table 2). Previous work showed that mouse retinal neurons exhibit cell-specific expression of the four different PMCA isoforms [32], however, this study did not examine differential PMCA expression in rod and cone synaptic terminals or present the IS results. A preliminary study with a 1:1000 dilution of the same anti-NCX1 rabbit polyclonal antibody we used at 1:100 dilution suggested that NCX was weakly expressed in rat cone, but not rod, photoreceptors and was more abundant in the inner retina [25]. However, no co-labeling experiments were performed to confirm this observation and there are no studies on NCX expression in mouse retina.

Overall, Figure 7A shows that pan-PMCA intensely labeled the synaptic terminals (plexiform layers) of the retina: as described with different PMCA antibodies [82,83]. In the outer retina, PMCA weakly labeled the IS and ONL and intensely labeled the OPL (Table 2). Double label experiments with VGluT1 and the selective cone antibody M-CAr [109] established the location of the large dome-shaped cone pedicles in the OPL (Figure 7B: purple pixels indicate colocalization). Figure 7C revealed that the intensely PMCA-positive rod spherules have a horseshoe-like or "V"-like [25] appearance. In contrast, the cone pedicles stained weakly and diffusely for PMCA (Figure 7C,H), except for a band around the distal part of the pedicle plasma membrane (Figure 7C: white arrowheads) and where the cone axon descends into the pedicle (Figure 7B: white arrowheads). Double labeling with PMCA and VGlut1 confirmed that PMCA extensively and uniformly labeled almost the entire rod spherule plasma membrane, whereas it only selectively labeled a region of the cone pedicle (Figure 7D: white arrowheads). Since PMCA2 labeled rod bipolar terminals in the IPL [32], we examined whether the dendrites in the OPL labeled with pan-PMCA. Pan-PMCA did not label the somas or dendrites of PKCα-positive rod bipolar cells, although PMCA and PKCα were in close apposition around the synaptic terminal region of the rod spherules (Figure 7E: yellow pixels).

In the inner retina, PMCA moderately labeled the INL, GCL, and Müller end feet (Figure 7A). Recently, it was shown that constant illumination increases Ca^2+^ in the proximal Müller glial cell processes [110]. Our results suggest that PMCA likely extrudes this Ca^2+^ from the Müller end feet. In addition, PMCA strongly labeled the entire IPL and more intensely labeled a band in each IPL sublamina (Figure 7A). These bands were identified previously as PMCA1-positive [32,67] and PMCA2-positive [32]. The location of the INL somas and dendrites of these cells, are similar to the PMCA1- and Ca^2+^ binding protein 5 (CaB5)-labeled cells identified as ON- and OFF-cone bipolar terminals [67]. In addition, the PMCA2-labeled cells [32] have a lamination pattern in the IPL similar to cholinergic amacrine cells [69,111].

Low and high magnification confocal images of retinas immunostained for NCX1, PMCA and/or peanut agglutinin (PNA) are presented in Figure 7F-K. NCX1 did not stain OS, showing that this NCX1 antibody did not cross-react with the ROS Na^+^/Ca^2+^-K^+^ (NCKX1) or COS (NCKX2) exchangers [112]. NCX1 intensely labeled IS, cone axons descending through the ONL (Figure 7F-K), the OPL (Figure 7G,J) and complete cone pedicles (Figure 7F-K,G,J: white arrows; Table 2). Weak to moderate labeling outlined somas in the INL and GCL. Diffuse labeling occurred throughout the IPL, although the IPL-α labeled more strongly than IPL-β. High magnification confocal images revealed that NCX1 diffusely labeled rod spherules (Figure 7G and Figure 7J). In cones, PMCA and NCX1 only colocalized in small bands at the top of the cone pedicle (Figure 7I and Figure 7K: yellow pixels): similar to that seen with M-CAr in Figure 7C.

In summary, rod spherule membranes intensely stained for PMCA, but only weakly and diffusely stained for NCX1. In contrast, cone pedicle membranes intensely stained for NCX1, but only the apical portion of the pedicle stained for PMCA. Additionally, NCX1, but not PMCA, labeled the RIS and CIS. These results confirm our hypothesis that the presynaptic Ca^2+^ transporters differentially distribute and localize in rod and cone photoreceptor terminals and strongly suggest that these differences have important functional implications relating to Ca^2+^ dynamics and neurotransmitter release.

**Figure 8:**
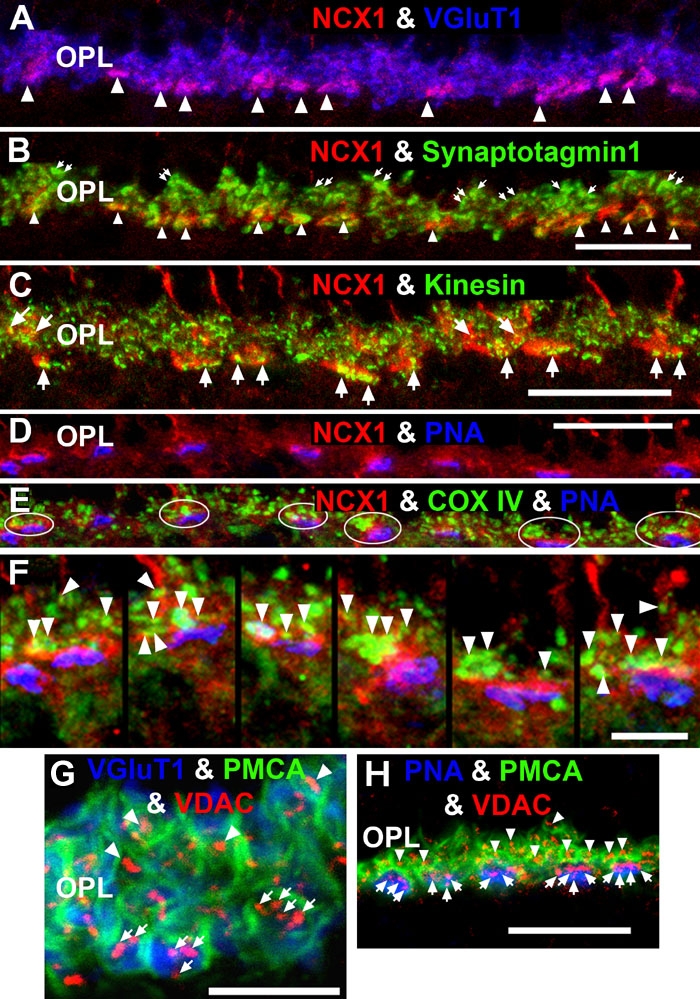
NCX1 localizes to ribbon synaptic units of cone pedicles and mitochondria closely associate with PMCA in photoreceptor terminals. **A**: NCX1-positive cone pedicles (red) and VGluT1 (blue) colocalize in the proximal ONL (white arrowheads: purple pixels). **B**: Synaptotagmin 1 (green) labels photoreceptor synaptic vesicles. Small NCX1- and synaptotagmin 1-positive puncta colocalize in rod spherules (white arrows: yellow pixels), while larger colocalized clusters are present in cone pedicles (white arrowheads: yellow-orange pixels). Scale bar equal 20 μm. **C**: Kinesin KIF3A (green) labels photoreceptor ribbons and docked synaptic vesicles. The kinesin-labeled rod spherules have an arc-shaped appearance and colocalize with diffusely located NCX1 (small yellow puncta). In contrast, cone pedicles have large clusters of double labeled NCX1- and kinesin-positive puncta (white arrows: yellow-orange pixels). Scale bar equal 20 μm. **D**-**F**: Mitochondria cluster away from the active zone in cone pedicles. Scale for **D** and **E** equal 20 μm and for **F** equal 10 μm. **D**: NCX1 (red) and PNA (blue) colocalize in cone pedicles (purple pixels). **E**: Triple labeling with NCX1, PNA and COX IV (green) reveals that the cone pedicles (white ellipsoids) contain multiple mitochondria that are located away from the ribbon synaptic unit. **F**: Higher magnification image of the same six pedicles in **E** reveals the COX IV and NCX1 colabeling (white arrowheads) and the distance of the COX IV-positive mitochondria from the active zone. **G**, **H**: Mitochondria closely associate with PMCA in the rod spherules and cone pedicles. Triple labeling with PMCA (green), VDAC (red), and with either VGluT1 (blue) or PNA (blue). Rods contain a single large mitochondrion (arrowheads) located close to the PMCA-labeled membranes. Cone pedicles contain multiple mitochondria (white arrows) clustered close to PMCA-labeled membranes, which are located away from the active zones. Scale bar equal 20 μm.

Based on their kinetic properties (i.e., Kd for Ca^2+^ and kcat) and the dependence of PMCA on ATP as a substrate, a second hypothesis was that NCX1 would be closely apposed to the active zones, whereas mitochondria would be closely apposed to PMCA in photoreceptor synaptic terminals. Figure 8A, a high magnification confocal image of a NCX1 and VGluT1 double label experiment, clearly shows that NCX1 intensely labeled cone pedicles (white arrows; purple pixels), but only diffusely labeled rod spherules. To determine whether NCX1 localized to the synaptic ribbon region (active zone), two different markers for rod and cone synaptic vesicles-synaptotagmin 1 [70,113] and kinesin KIF3A [71] were used in colocalization experiments. Figure 8B shows that cone pedicles had large clusters of double labeled NCX1- and synaptotagmin 1-positive puncta (white arrowheads: yellow-orange pixels), whereas only small NCX1- and synaptotagmin 1-positive puncta were present in rod spherules (white arrows: yellow pixels). The kinesin KIF3A-labeled rod spherules had an arc-shaped appearance (Figure 8C), consistent with the strong kinesin KIF3A labeling of the photoreceptor ribbon matrix [71]. When retinas were double labeled with NCX1 and kinesin the cone pedicles had large visible clusters of double labeled NCX1- and kinesin-positive puncta (white arrows: yellow-orange pixels), whereas rod spherules contained only small yellow puncta. Double labeling with PMCA and synaptotagmin1 (data not shown) or syntaxin 3 [32] revealed that PMCA did not colocalize with either marker, indicating that PMCA was not present at the active zone of rod spherules.

**Figure 9:**
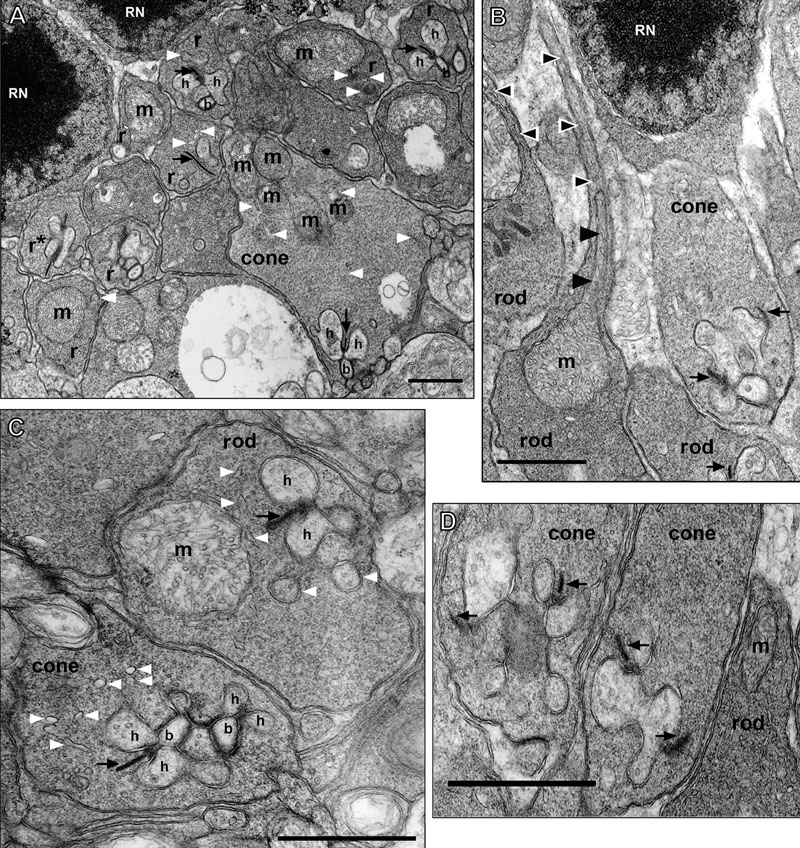
Ultrastructure of rod and cone photoreceptor synaptic terminals. **A**: The rod spherules (r) form two to four tiers that overly the larger more electron lucent cone pedicles (cone). Two rod nuclei (RN) are present. The rod spherules contain a single large mitochondrion (m), whereas the cone pedicles contain several mitochondria. Cisterna of smooth endoplasmic reticulum (white arrowheads) are often located adjacent to individual mitochondrion. Both terminals are filled with round synaptic vesicles. The invaginating processes of two lateral horizontal cells (h) and a central bipolar cell (b), the classic triad [122], are seen at the short synaptic ribbon (black arrow) of the cone and the longer synaptic ribbon (black arrow) of the rod in the upper right corner of the figure. The rod spherule on the left labeled r* appears to be a rod with two synaptic ribbon units as described [121]. **B**: Rod spherule and axon; cone pedicle. On the left portion of the panel, a descending rod axon (black and white arrowheads) and its expansion into a rod spherule (rod) is visible for two rods. An elongated mitochondrion (m; black arrowheads) is visible in the thin axon (0.20-0.25 μm) as it enlarges into the spherule. The rod spherules are filled with 25-35 nm electron dense round synaptic vesicles and a single large mitochondrion. A tangential view of an adjacent rod spherule with a synaptic ribbon (black arrow) is shown. A larger more electron lucent cone pedicle has two visible synaptic ribbons (black arrows) at invaginating synapses in this section. A rod nucleus (RN) in the most proximal row of the ONL is present. **C**: Rod spherule and cone pedicle. The rod spherules contain a single large mitochondrion (m) and an invaginating process with two lateral horizontal cells (h) and a central bipolar cell that is just out of the section. The rod has a long synaptic ribbon (black arrow) and a visible arciform density at its base. The most proximally located cone pedicle has two classic triads visible with two lateral horizontal cells (h) and a central bipolar cell (b). Other horizontal cell processes also are visible and the arciform density is evident. Cisterna of smooth endoplasmic reticulum (white arrowheads) are more numerous in cone pedicles than rod spherules. **D**: Two cone pedicles and a rod spherule. Each large cone pedicle has multiple synaptic ribbons (black arrows) at invaginating synapses. Several lateral horizontal cell processes, of varying size and intensity, are visible. The enlarged axonal region of rod spherule, with its darker matrix, contains a mitochondrion (m) that is just out of the section. Scale bars for all panels equal 1 μm.

A set of triple labeling confocal experiments determined the spatial interrelation of synaptic terminal mitochondria to NCX1-labeled (Figure 8D-F) and PMCA-labeled (Figure 8G,H) synaptic membranes. Triple labeling with NCX1, PNA and COX IV revealed that cone pedicles contained multiple mitochondria that were not located at the ribbon synaptic unit (Figure 8E: white ellipsoids). In high spatial resolution images, it is evident that the mitochondria in the same six cone pedicles are located relatively far from the active zone (Figure 8F: white arrowheads). Triple labeling experiments with PMCA, VDAC and VGluT1 (Figure 8G) or PMCA, VDAC and PNA (Figure 8H) suggest that rods contain a single large mitochondrion (white arrowheads) located close to the horseshoe-shaped PMCA-labeled synaptic membranes. In contrast, the cone pedicles contain multiple mitochondria (white arrows). However, the mitochondria in cone pedicles cluster close to the PMCA-labeled membranes located near the apical portion of the pedicles. As described below in more detail, electron microscopic studies confirmed these observations (Figure 9).

In summary, these results confirmed our second hypothesis that NCX1 localizes to active zones, whereas mitochondria are closely apposed to PMCA in photoreceptor synaptic terminals. These results expand our understanding of how the two major presynaptic Ca^2+^ transporters can differentially regulate rod spherule and cone pedicle Ca^2+^ levels during sustained depolarization in darkness and thereby participate in the kinetic regulation of neurotransmitter (glutamate) release. Our high-resolution immunocytochemical results on the presynaptic localization of NCX1 and PMCA in photoreceptors are not entirely consistent with the epifluorescent microscopy study of the rat retina, which states that NCX1 staining was weak in rat cones and absent in rods [25]. However, this difference is understandable since 10 fold less NCX antibody was used in the rat study, which resulted in no NCX labeling in the IS and very weak NCX labeling in the OPL [25].

### Cone pedicles have more ATP production capacity than rod spherules: conventional electron microscopy and tomography of mouse outer plexiform layer and photoreceptor synaptic terminal mitochondria

**Figure 10:**
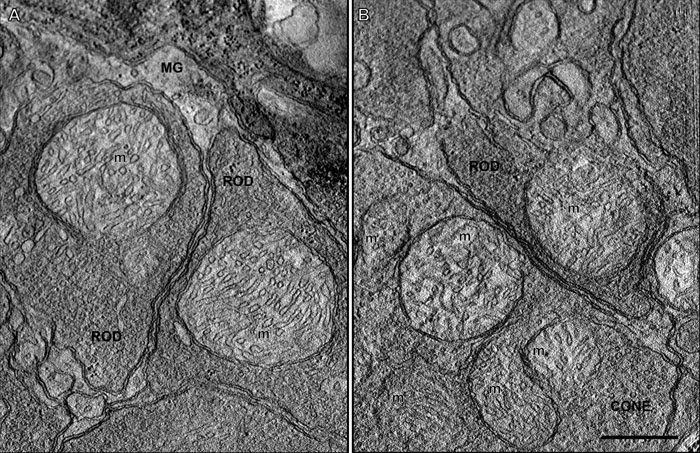
A 2.2 nm thick slice through a rod spherule and cone pedicle mitochondrion from three-dimensional electron tomographic reconstructions. The rod and cone mitochondria (m) are in the orthodox conformation, revealing that they were fixed and preserved in their non-energized state [55,123]. **A**: Two rod spherules present in the distal outer plexiform layer. Each rod spherule contains only one large almost circular mitochondrion. Müller glial processes (MG) encircle the rod spherules. **B**: A rod spherule overlying a cone pedicle in the outer plexiform layer. The larger cone pedicle contains five mitochondria (m). Scale bar equal 500 nm.

A number of electrophysiological, biochemical and morphological observations indicate that cone synaptic terminals, compared to rods, have a higher ATP demand [18,47,114-120]. To test the functionally-based hypothesis that the ATP production capacity of cone pedicles is greater than that of rod spherules, we used EM and ET to examine the number, size, volume and total inner membrane system surface area of mitochondria [42,43] in the synaptic terminals of rods and cones. The high magnification electron micrographs of rod and cone synaptic terminals, presented in Figure 9A-D, reveal numerous important morphological differences between rod spherules and cone pedicles and their associated mitochondria. First, Figure 9A illustrates a fundamental organizational feature of the OPL. That is, three to five tiers of rod spherules are located above the larger more electron lucent cone pedicles (also see Figure 4). Second, rod spherules contained a single very large ovoid mitochondrion, as described [28]. It is especially worth noting that the rod mitochondrion is located close to the synaptic ribbon complex (Figure 9A,C and Figure 10), consistent with our LCSM studies. Second, cone pedicles have four to six closely grouped mitochondria (per pedicle±SEM=5.2±0.2) that are located in the distal portion of the pedicle relatively far from the synaptic ribbon complexes (Figure 9A and Figure 10B): as shown in the confocal studies (Figure 8). This is reminiscent of the goldfish retinal Mb1 cell where multiple mitochondria cluster away from the active zone [13]. Third, the rod spherule mitochondrion is larger (1.8-2.0 μm diameter) than any of the cone pedicle mitochondria (0.8-1.5 μm diameter). The rod mitochondrion occupies 25-30% of the spherule volume, whereas the cone pedicle mitochondria collectively occupy 10-15% of the pedicle volume (Figure 9A-C and Figure 10). Fourth, the average volume of the rod spherule is 3.0-3.5 μm^3^, whereas the average volume of the cone pedicle is about 10 times larger. Fifth, rod spherules usually contain one ribbon synapse (Figure 9A-C). It was estimated that about 5% contain two ribbons [120,121], as illustrated in the rod spherule labeled r* in Figure 9A. Occasionally, we noticed that the most proximal rod nuclei had a ribbon synapse at the base of its soma, as described [94], and the spherule lacked a mitochondrion (data not shown). Sixth, cone pedicles contain 6 to 14 synaptic ribbons (Figure 9B-D [52,53]; data not shown). The invaginating processes of two lateral horizontal cells (h) and a central bipolar cell (b), the classic triad [122], were seen at the short synaptic ribbon of the cone and the longer synaptic ribbon of the rod (Figure 9A). The arciform density was noticeable at the base of each of several ribbons.

In addition to their differences, rod and cone pedicles and their mitochondria share some similarities. First, the mitochondria in rod and cone synaptic terminals were in the orthodox conformation (Figure 10). This configuration is characterized by a relatively large matrix volume and small intracristal space, as the mitochondrial IBM is closely apposed to the OMM [123]; Figure 1. Second, mitochondria in both the spherules and pedicles are relatively large compared to the IS mitochondria [55] and those in other neural tissues [89,93,124], although the rod spherule mitochondria are larger and rounder. Third, cisternae of endoplasmic reticulum (ER) were observed near the mitochondria and synaptic ribbon of both the rod spherule and cone pedicle (Figure 9A,C). In some instances, the ER was likely contiguous with or closely apposed to individual mitochondrion, as observed in rat spherules [28]. ER also were observed in frog rod and cone synaptic terminals [29,30], where ATP markedly enhanced the Ca^2+^ uptake by rod spherule ER [29].

### Analysis of the three-dimensional structural features of rod spherule and cone pedicle mitochondria

**Figure 11:**
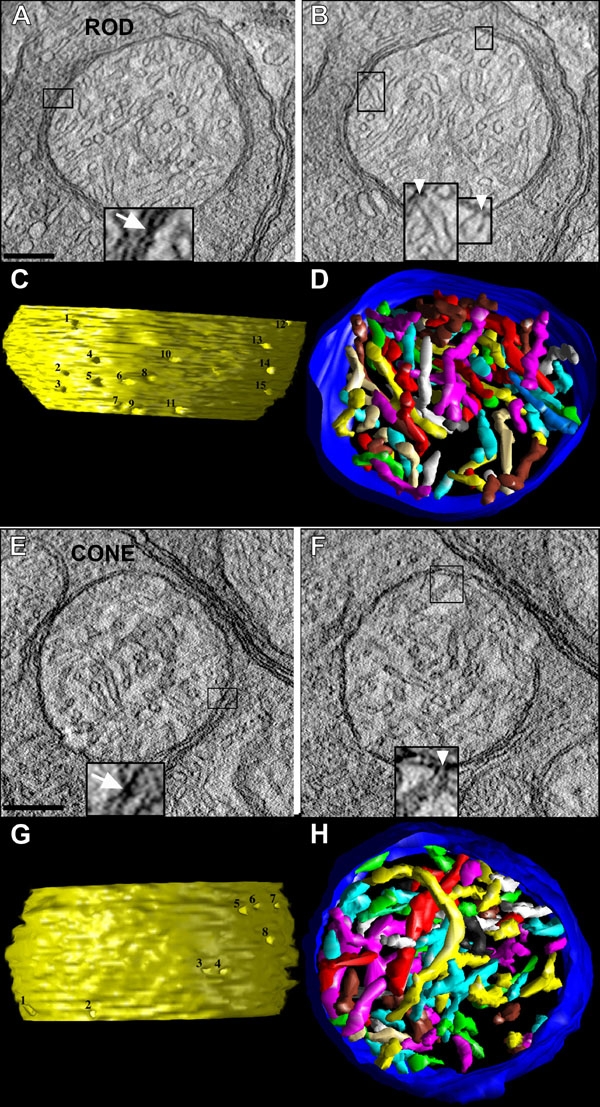
Three-dimensional electron tomograms of a rod spherule and a cone pedicle mitochondrion. **A**-**D**: Three-dimensional imaging of a rod spherule mitochondrion. **A**: A representative 2.2 nm slice through a tomographic volume of a rod spherule (from Figure 9A) that shows a large mitochondrion with many cristae. The inset (enlarged 3X) illustrates an example of a classical contact site, defined as the location where the OMM and IBM join (arrow). Scale bar for **A** and **B** equal 200 nm. **B**: Another slice through the volume showing two crista junctions (CJs: boxed) that have tubular openings, which connect the cristae with the intermembrane space. The inset at the bottom shows the openings (white arrowheads) of the two CJs enlarged 2X. **C**: Side view of the inner membrane of the segmented volume displayed with left lighting. CJ openings are invariably narrow, tubular and remarkably uniform in diameter. There are fifteen numbered CJ openings in this view. **D**: Top view of the segmented volume showing the outer membrane (blue) and a subset of cristae (various colors). Most of the cristae are tubular. However, some cristae possess lamellar compartments, which connect to the intermembrane space via CJs. **E**-**H**: Three-dimensional imaging of cone pedicle mitochondrion. **E**: A representative 2.2 nm slice through a tomographic volume of a cone pedicle (from Figure 9B) that shows a typical medium- to large-sized mitochondrion typical at this terminal. As illustrated, the abundance of cristae membranes is significantly smaller in each cone pedicle, compared to rod spherule, mitochondria. The inset (enlarged 3X) illustrates an example of a classical contact site (arrow). Scale bar for **E** and **F** equal 200 nm. **F**: Another slice through the volume showing two CJs (boxed). The inset at the bottom shows the openings (white arrowhead) of the two CJs expanded 2X. The openings of these crista junctions are significantly smaller than their counterparts in the rod spherule mitochondria (Table 3). **G**: Side view of the inner membrane of the segmented volume displayed with left lighting. The eight CJ openings seen in this view are numbered. **H**: Top view of the segmented volume showing the outer membrane (blue) and a subset of cristae (various colors). Most of the cristae are tubular, as in the rod spherule mitochondrion, although a small proportion have lamellar compartments. The cristae surface area and volume are significantly smaller in cone pedicle, compared to rod spherule, mitochondria (**B**, **C**).

**Figure 12:**
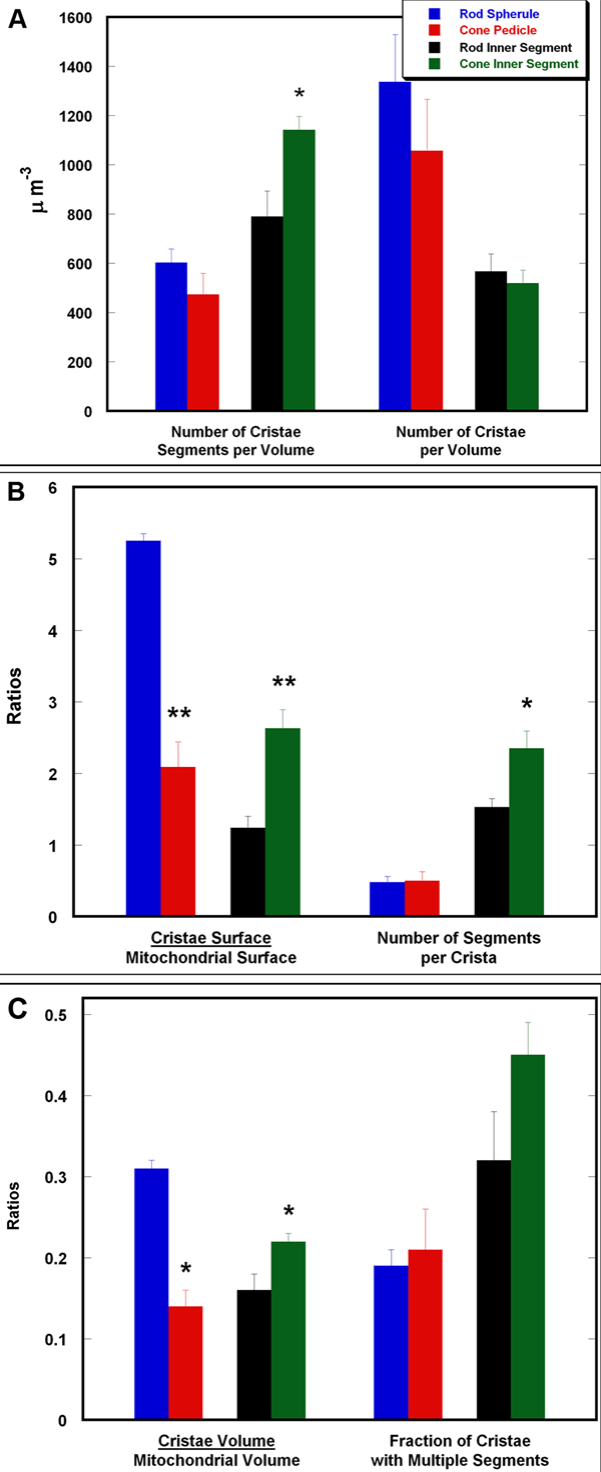
Cristae measurements of rod spherule and cone pedicle mitochondria obtained from tomographic reconstructions. **A**-**C**: Comparison of cristae measurements in rod and cone synaptic terminal and inner segment mitochondria. The reconstructed portion of the mitochondrion was segmented along membranes: outer membrane, inner boundary membrane and cristae membranes. The programs Synuarea and Synuvolume calculated the volume and surface area values for the outer membrane (used for "mito" in the denominator) and each crista. The values for individual cristae were summed to provide the number per volume (**A**) or numerator of the ratios (**B** and **C**). The mean values (±SEMs) for mitochondria in rod spherules (blue bars) and cone pedicles (red bars) are presented in each plot. In addition, our previously published mean (±SEMs) values for rod inner segment (black bars) and cone inner segment (green bars) mitochondria are plotted for ease of comparison [55]. Measurements were conducted on the tomographic reconstructions from three rods and three cones from three different mice. A two-tailed Student's t-test determined significant differences: *p<0.05; **p<0.01.

To determine the total inner membrane system surface area of mitochondria, a quantitative analysis and comparison of the ultrastructure and substructure of rod spherule and cone pedicle mitochondria using the high-resolution 3-dimensional tools of ET was required (Figure 11 and Figure 12; Table 3). Since there is a paucity of detailed structural information about rod spherule and cone pedicle mitochondria, another goal was to compare and contrast the structural features of photoreceptor synaptic terminal mitochondria with our published ultrastructural and substructural results of mouse RIS and CIS mitochondria [40,55,56]. Volume segmentation provided an analysis of individual cristae. Their 3-dimensional shapes and membrane architecture were visualized in varying orientations to classify the structural motifs of lamellae, tubes, and constrictions (narrow connections between crista segments) per each crista. Surface rendering and measurements of volume and surface elements permitted a comparison of substructures inside mitochondria (Figure 12; Table 3).

**Table 3:** Mitochondrial crista junction diameter in rod and cone photoreceptors and brain regions.

**Tissue or cell type**	**Number of measurements**	**Mean crista junction diameter (nm)**	**Standard deviation (nm)**	**Reference**
Rod Spherule	102	12	4	this paper
Cone Pedicle	32	9	2	this paper
Rod Inner Segment	212	17	6	40,55
Cone Inner Segment	31	12	4	55
Cerebellum	85	16	5	93
Striatum	43	14	4	93
Hippocampus	38	14	4	93
Cortex	26	14	5	93

The tissue or cell type, number of measurements, mean crista junction diameter±SD, and reference are presented.

Three-dimensional ETs revealed that rod spherule and cone pedicle mitochondria contain a very large number of cristae (Figure 11 and Figure 12). The mean±SEM number of cristae per mitochondrial volume for rod spherules and cone pedicles was 1,337±192 and 1,057±209 μm^-3^, respectively. The cristae, presented after segmentation and surface rendering, are represented by different colors (Figure 11D,H). Most of the cristae in rod and cone synaptic terminal mitochondria were tubular, however, a few had lamellar compartments. Although the rod spherule mitochondria had 26% more cristae per unit volume than the cone pedicle mitochondria, this was not statistically different (Figure 12A). However, there were significantly more cristae membranes in a rod spherule, compared to a cone pedicle, mitochondrion. That is, the cristae surface/mitochondrial surface (i.e., amount of cristae membranes) and cristae volume/mitochondrial volume were 2.2-2.5 fold larger in the rod spherule mitochondrion, compared to a cone pedicle mitochondrion (Figure 12B,C). Overall, and importantly, the four to six cone pedicle mitochondria collectively contain twice as many cristae membranes as the single rod spherule mitochondrion. In contrast, the number of cristae segments per volume (Figure 12A), number of segments per crista (Figure 12B) and fraction of cristae with multiple segments (Figure 12C) were similar in rod spherule and cone pedicle mitochondria. A detailed examination of Figure 12 reveals that there are distinct and marked differences in the rod and cone cristae of mitochondria located in the synaptic terminal compared to those in the inner segment [55].

There are two classes of contact sites: classical and bridge. The classical contact site is defined by the OMM and IBM pinching together (Figure 1), whereas the bridge contact site spans across the intermembrane space without a change in distance between the outer and inner membranes [55,93,125]. Tomographic reconstructions showed that both types of contact sites were present in rod spherule and cone pedicle mitochondria. A crista junction (CJ) is the site where a crista connects to the IBM [89,126]. CJs are tubular openings that are invariably narrow in orthodox mitochondria, remarkably uniform in diameter, and connect the intracristal space with the intermembrane space (Figure 11B-D and Figure 10F-H). The de novo formation of tubular CJs is energetically favorable as these are dynamic structures [127]. The average CJ diameter in brain mitochondria is 14-16 nm, whereas it is 12 nm in CIS mitochondria and 17 nm in RIS mitochondria [55,93] (Table 3). The usual narrow opening and uniform size of the CJ likely restricts the diffusion of cytochrome c and ADP/ATP out of the intracristal compartments, adjacent to the membranes where the respiratory complexes are concentrated [124,126,128]. Unexpectedly, the mean CJ in cone pedicle mitochondria was 9 nm (Figure 11F). This is significantly smaller than the 12 nm diameter measured in rod spherule mitochondria (Figure 11B) and smaller than any other known CJ (Table 3).

In summary, these novel ET results revealed that rod and cone synaptic terminal mitochondria share some unique substructural features that are different from mitochondria in the rod and cone inner segments (Figure 12) and other neurons (Table 3). In addition, the rod and cone synaptic terminal mitochondria possess significantly different and uniquely distinguishing characteristics (Figure 12; Table 3).

### Ca^2+^ imaging and correlative electron microscopy of rod and cone synaptic terminals in dark-adapted and light-adapted whole retinas

**Figure 13:**
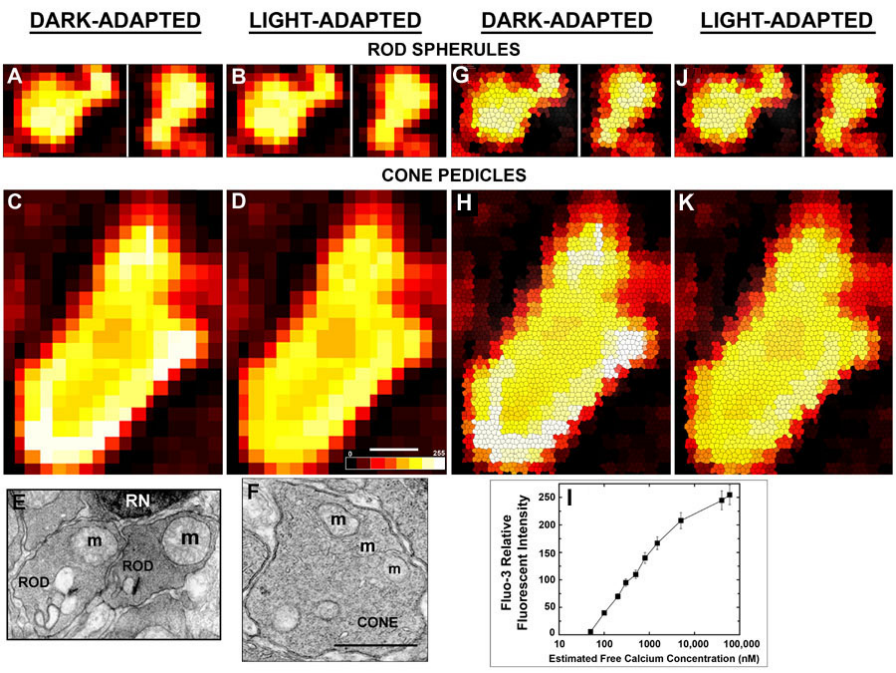
Ca^2+^ imaging and correlative electron microscopy of rod and cone synaptic terminals in dark-adapted and light-adapted retinas. **A**-**D**: Pseudocolored Ca^2+^-fluo-3 confocal images obtained from an adjacent pair of rod spherules (**A**, **B**) and a single cone pedicle (**B** and **D**) during dark adaptation (**A** and **C**) and light-adaptation (**B** and **D**). Scale bar equal 1 μm. The pseudocolor scale represents pixel intensity from 0 to 255 gray levels. The RFI values and estimated free [Ca^2+^] in dark-adapted rod spherules were lower than in cone pedicles, whereas the RFI values and estimated free [Ca^2+^] in light-adapted cone pedicles were markedly lower than in light-adapted rod spherules (Table 4). During light adaptation, the cone pedicles lowered their intraterminal [Ca^2+^] about 3 fold more than rod spherules (Table 4). **G**-**K**: For higher resolution and better visualization of the Ca^2+^ microdomains, the pseudocolored images were transformed using an advanced image synthesis algorithm. The microdomains of high [Ca^2+^] were larger and closer to the plasma membrane in dark-adapted cone pedicles (**C** and **H**) compared to rod spherules (**A** and **G**). The scale bar and pseudocolor scale are as presented in **D**. **E** and **F**: The electron micrographs of the rod spherules **E** and cone pedicle **F** correspond to the rod spherules in panels A and B and cone pedicle in **C** and **D**, respectively. RN: rod nuclei, m: mitochondrion. Scale bar equal 1 μm for both panels. **I**: A fluo-3 Ca^2+^ calibration curve used to estimate the free [Ca^2+^] in the photoreceptor synaptic terminals (see Table 4).

The above results, in concert with the demonstrated sensitivity and kinetics of rod and cone synaptic transmission [9,17-21], predict that the free [Ca^2+^] will be lower in dark-adapted mouse rod spherules compared to cone pedicles. In addition, they suggest that mouse cone pedicles would exhibit faster and larger spatiotemporal decreases in free [Ca^2+^] during light adaptation than rod spherules. To test these two functionally-based hypotheses, fluo-3 Ca^2+^ imaging and LSCM experiments were conducted in retinal slices during darkness (Figure 13A,C) and one min after the onset of a rod saturating stimulus (Figure 13B,D). This time period was used because the light-adapted decrease in photoreceptor oxygen consumption stabilizes during the first minute of light stimulation [49,50,101]. Figure 13 presents representative pseudocolored images of free [Ca^2+^] from two adjacent rod spherules (Figure 13A,B) and a nearby cone pedicle (Figure 13C,D). For higher resolution and better visualization of the Ca^2+^ microdomains, the pseudocolored images were transformed using an advanced image synthesis algorithm (Figure 13G-K). The corresponding electron micrographs of these rod spherules and cone pedicle are shown in relatively the same orientation (Figure 13E,F). Considering the various preparation procedures and length of time that the retinas were incubated in different buffers, the mitochondria and synaptic terminals are intact and relatively well preserved compared to our immediately fixed tissue (Figure 9). In addition, the Ca^2+^ and fluo-3 calibration curve, used to estimate free [Ca^2+^] in the photoreceptor synaptic terminals, is presented in Figure 13G.

Table 4 presents the Ca^2+^-fluo-3 RFI values and the estimated mean free [Ca^2+^] for the dark- and light-adapted rod spherules and cone pedicles. The estimated free [Ca^2+^] in dark-adapted rod spherules was 3.2 fold lower (about 2.2 μM) than in cone pedicles (about 6.8 μM), which is consistent with our hypothesis that dark-adapted rod spherules maintain a lower mean resting [Ca^2+^] than cone pedicles. In contrast, the estimated free [Ca^2+^] in light-adapted cone pedicles was 3.2 fold lower (about 0.2 μM) than in light-adapted rod spherules (about 0.7 μM). Thus, during light adaptation the estimated free [Ca^2+^] decreased 2 fold in rod spherules and almost 30 fold in cone pedicles compared to darkness. These latter results reveal that during light adaptation cone pedicles rapidly and efficiently lowered their intraterminal [Ca^2+^] compared to rod spherules. This is consistent with our immunocytochemical, EM and ET findings that cone pedicles possess structural and functional mechanisms for the more rapid removal of intraterminal free Ca^2+^ than rod spherules (vide supra).

**Table 4:** Ca^2+^-Fluo-3 relative fluorescence intensity values in dark-adapted and light-adapted rod and cone photoreceptor synaptic terminals.

	**Dark-adapted**	**Light-adapted**
Rod Spherules	180.5±4.9	138.2±6.7
Cone Pedicles	214.8±9.7	82.7±4.6

All mean±SEM relative fluorescence intensity values are scaled from 0 to 255 as described in the Materials and Methods section and as shown in Figure 13.

The microdomains of high [Ca^2+^] were larger and closer to the plasma membrane in dark-adapted (depolarized) cone pedicles (Figure 13C,H) compared to rod spherules (Figure 13A,G). In dark-adapted retinas, we estimated that the Ca^2+^ microdomains were 300-400 nm in rod spherules (Figure 13A) and 600-700 nm in cone pedicles, where they overlapped in the vicinity of the synaptic ribbons (Figure 13C). Furthermore, the RFI appeared higher in the presumed location of the rod spherule mitochondria than in the cone pedicle area where mitochondria cluster. This suggests that rod mitochondria maintain a higher matrix [Ca^2+^] than cone mitochondria, which is consistent with the location of rod spherule mitochondria and our suggestion that rod spherule mitochondria actively participate in intraterminal Ca^2+^ buffering.

The presynaptic terminals of the bullfrog saccular and axolotl lateral-line hair cells contain dense bodies that reversibly bind fluo-3 with a Kd of 550 nM [129]. We conducted high magnification electron microscopy studies on dark-adapted mouse retinas to determine whether the Ca^2+^ microdomains in our dark-adapted photoreceptor synaptic terminals might be due to the binding of fluo-3 to presynaptic dense bodies. Consistent with the findings of Vollrath et al. [130], we did not see any dense spheres in the photoreceptor terminals of our dark-adapted mouse retinas. Thus, we conclude that the Ca^2+^ microdomains do not result from fluo-3 binding to this synaptic organelle.

## Discussion

This study tested several functionally-based hypotheses related to the metabolic coupling and cross-talk between rod and cone synaptic terminal mitochondria, ER, PMCA and NCX and their potential roles in generating/using ATP and regulating Ca^2+^ dynamics for neurotransmitter release. Three novel and significant sets of results were obtained. First, we characterized the spatial interrelation between mitochondria, ER, Ca^2+^ transporters and active zones in ribbon synapses as well as determined these details in the retina and photoreceptor synaptic terminals. Second, we determined the distribution, number and structure of mitochondria in rod spherules and cone pedicles as well as their substructure by utilizing ET. Third, we determined the mean levels of free [Ca^2+^] in rod and cone synaptic terminals of whole isolated retinas during darkness and light adaptation.

**Figure 14:**
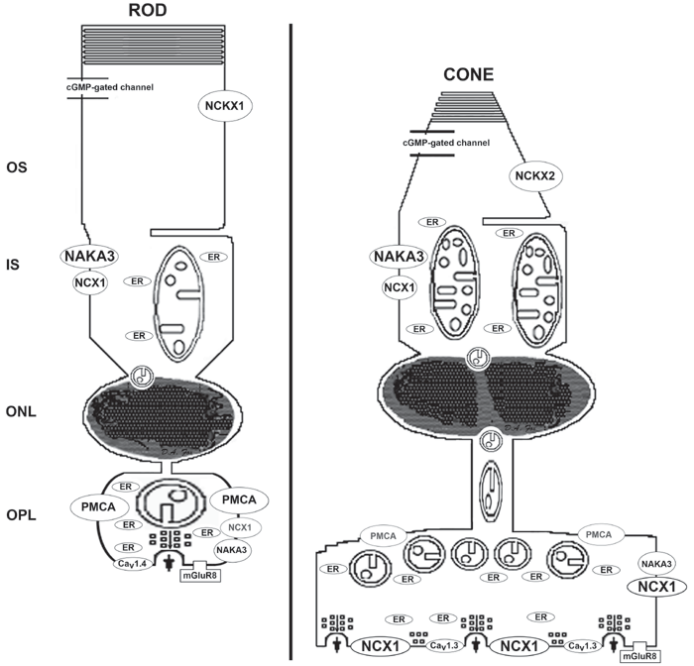
Summary diagram of the mouse rod and middle wavelength (M) cone ribbon synapses. The diagram summarizes our current findings, includes results from our work on mouse rod and cone photoreceptor inner segment (IS) mitochondria [55], and highlights the results from numerous other investigators. It compares the location, distribution and morphology of the major cellular components involved in regulating ATP and Ca^2+^ homeostasis, and presynaptic glutamate release. The size and/or color of the lines, ellipses, letters and mitochondria reflect differences in activity determined by histochemistry or electrophysiology, protein density as visualized with immunolabeling, and number or morphology as determined electron microscopy and electron tomography. The relatively thicker lines and larger ellipse in the cone outer segments (OS), compared to ROS, indicate the cone's higher relative permeability for Ca^2+^ through the cGMP-gated channel, higher fraction of the dark current carried by Ca^2+^ and more rapid Na^+^/Ca^2+^-K^+^ exchanger (NCKX2 in cones compared to NCKX1 in rods) [59-61,112,184]. RIS have an average of about 5 mitochondria per cross sectional area, whereas CIS have about 10 mitochondria per cross sectional area resulting in a cone to rod ratio of 2:1 [55]. The mouse RIS and CIS mitochondria are in the orthodox, rather than the condensed, conformation [55]. The mean cristae junction diameter of the RIS and CIS mitochondria is 17 and 12 nm, respectively [55]. CIS mitochondria are more uniformly stained and reactive for cytochrome c oxidase than RIS mitochondria [55]: depicted here as darkened inner mitochondrial membrane and cristae. Both RIS and CIS contain numerous small calreticulin- and SERCA3-positive smooth endoplasmic reticulum cisternae/vesicles (Figure 6, Figure 9B,D): several are closely associated with mitochondria (Figure 6B,E): herein labeled ER. Photoreceptor IS are weakly immunoreactive for PMCA (Figure 6A). NCX isoform 1 (NCX1) immunolabeling was intense in both RIS and CIS, although it is stronger in the latter (Figure 7D). In monkey retina, CIS had more intense immunolabeling for Na^+^,K^+^-ATPase (NAKA) than RIS [40]: likely the NAKA α3 isoform (NAKA3) [101,167]. Similar data is not yet available in the mouse retina. In the most proximal rows of the ONL, a juxtanuclear mitochondrion sits above individual rod nuclei (Figure 2A,D and Figure 4). In the most distal ONL, a pair of previously undescribed juxtanuclear mitochondria localizes above and below the cone nuclei (Figure 2-Figure 4). The rod spherule contains one large mitochondrion. Cone pedicles have about 5 mitochondria per cell resulting in a cone to rod ratio of 5:1 (Figure 9 and Figure 10). The synaptic terminal mitochondria of rods and cones are in the orthodox or energized conformation (Figure 10). The mitochondria in the cone pedicle cluster near top of the terminal, where the axon enters the pedicle (Figure 7, Figure 8D-E, Figure 9B, and Figure 10B). The mean cristae junction diameter of the rod spherule and cone pedicle mitochondria is 12 and 9 nm, respectively, (Table 3). Mouse cone pedicles are more reactive for cytochrome c oxidase than rod spherules (same style depiction as above; Fox, unpublished data), as described for other mammalian retinas [47,62]. Rod and cone synaptic terminals contain numerous small calreticulin- and SERCA3-positive smooth endoplasmic reticulum cisternae/vesicles (small gray ellipses): several are closely associated with mitochondria (Figure 6, Figure 9B,D). Rod spherules intensely label for PMCA, whereas cone pedicles exhibit minimal labeling that localizes at the top of the pedicle (Figure 7B-C, Figure 8D,E). Cone pedicles label intensely and uniformly for NCX1, whereas rod spherules label weakly and more diffusely (Figure 7D-F and Figure 8). Rod spherules have one synaptic invagination (Figure 9), although about 5% had two ribbon synaptic units (Figure 9B) as described [153,185]. Cone pedicles have 6-14 synaptic invaginations (Figure 8: Fox, unpublished data). In the dark-adapted mouse retina, Ca^2+^ enters rod and cone synaptic terminals through voltage-gated L-type calcium channels Ca_v_1.4 (formerly α1F) and Ca_v_1.3 (formerly α1D), respectively [160,161] and glutamate is continuously released. Moreover, in the dark-adapted mouse retina the rod spherules maintain a significantly lower free [Ca^2+^] than cone pedicles (Figure 13; Table 4). In contrast, during light adaptation the cone pedicles more rapidly, efficiently and completely lower their free [Ca^2+^] compared to the rod spherules (Figure 13; Table 4). The group 3 metabotropic glutamate receptor mGluR8 is present on the presynaptic terminals of rod and cone photoreceptors [186]. It likely provides negative feedback control on glutamate release and serves to prevent Ca^2+^ overload [39,40,94] by down-regulating the intraterminal Ca^2+^ levels [186]. The implications of the above findings related to rod spherule and cone pedicle bioenergetics, Ca^2+^ homeostasis, apoptosis and neurotransmission are discussed in the text.

Overall, our LSCM results revealed that retinal mitochondria exhibit laminar, cellular and compartmental segregation in different retinal neurons: see Table 2 and schematic summary Figure 14. For example, a strong-intense COX IV staining pattern was evident in mitochondria located in IS, OPL, IPL-α, IPL-β, GCL and Müller glial cell end-feet, whereas the INL only had weak-moderate COX IV labeling. The photoreceptors displayed three separate compartments of strong to intense COX IV labeling: IS, portions of the ONL, and the OPL. The CIS labeled more intensely for COX IV than the RIS, consistent with their two-fold higher mitochondrial content [55]. In contrast, bipolar cells exhibited differential staining such that there was moderate COX IV staining in their somas while the terminals labeled strongly. The intense labeling of IPL-α (ON-sublamina) and IPL-β (OFF-sublamina) with all three mitochondrial markers likely reflects the high amount of ATP required for glutamate uptake into synaptic vesicle and synaptic vesicle priming at ribbon synapses [1,33,34]. The above observations are consistent with functional results showing that mitochondrial oxygen consumption is higher in photoreceptors than in the inner retina during darkness and illumination [49,97] and that the mean inner retinal oxygen consumption is similar during dark and light adaptation [49,50,101]. In addition, the COX IV and POLG staining patterns were coincident in all retinal layers, except the INL. This suggests that mitochondria located in regions of high metabolic demand, where they might readily produce reactive oxygen species and initiate apoptosis, also might have an increased capacity for mitochondrial DNA replication and repair.

The overall VDAC immunolabeling pattern was similar to that of COX IV and POLG: illustrated by the areas of COX IV and VDAC colocalization. However, VDAC only weakly-moderately labeled IS. This lower IS immunofluorescence, using the pan-VDAC antibody, is not due to antibody specificity or tissue penetration problems, since this antibody recognizes a conserved sequence in all three VDAC isoforms (BLAST) and intensely stained mitochondria in the OPL. The weaker VDAC labeling might result from a low concentration of ER in the IS, since VDAC is found in ER closely apposed to mitochondria [131]. However, this is not likely since the calreticulin, SERCA3 and EM findings reveal an extensive amount of ER in RIS and CIS. Thus, VDAC protein expression is relatively low in mouse IS: a conclusion supported by in situ hybridization results [132]. This raises the intriguing possibility that lower levels of VDAC2 in IS and particularly RIS, increase the susceptibility of rods to mitochondrially mediated apoptosis [40,94,133] as VDAC2 inhibits BAK activation and apoptosis [134]. Finally, the strong to intense VDAC labeling and colocalization with VGluT1 in the plexiform layers suggests another possible level of metabolic coupling between mitochondria and synaptic transmission, since physiological concentrations of glutamate modulate VDAC channel activity [135].

During the LSCM and EM studies, we unexpectedly found distinct and highly localized COX IV-, VDAC- and POLG-positive mitochondria associated with cone and rod nuclei. First, in the most distal ONL large juxtanuclear mitochondria were located above and below the cone nuclei. The mitochondrion below the cone nucleus is in the axon, as described [53]. Second, in the most proximal ONL numerous single, smaller juxtanuclear mitochondria were situated above each rod nucleus. It is likely that these juxtanuclear mitochondria are involved with somal ATP synthesis, phosphotransfer networks and nucleocytoplasmic communication [136]. For example, cone nuclei contain larger amounts of electron lucent, transcriptionally active euchromatin compared to rods ([53]; Figure 3 and Figure 8A). This suggests that transcription and other ATP-requiring nuclear processes [136,137] are more active in cones than rods. In addition, cone nuclei are located in the most distal portion of the ONL, such that their somas are further from their synaptic terminals than those of rods. Moreover, glycolytic and glucose-6-phosphate dehydrogenase enzyme activities are low in the ONL compared to other photoreceptor compartments [138] and ATP has a short diffusion distance [139]. Thus, local ATP synthesis is particularly important for these cone nuclei. The proximal rod somas likely are dependent upon these juxtanuclear mitochondria because they lack a classic spherule that contains a single mitochondrion, although they possess a ribbon synapse and active zone at the base of their somas [122]. Interestingly, these rod juxtanuclear mitochondria and somas appear selectively vulnerable to Ca^2+^ overload or oxidative stress [39,140,141].

The mechanisms and molecular signals that direct retinal mitochondria to their different cellular and compartmental sites are unknown. Neuronal mitochondria, compared to other organelles, have several unique properties with respect to axoplasmic transport. First, they undergo both anterograde and retrograde transport. Second, their motility is characterized by saltatory movements and prolonged stationary phases. Third, the net direction of movement varies with the physiological status of the axon. Fourth, mitochondria utilize microtubules and actin-myosin based microfilament systems for motility, and neurofilament associations for static phases within axons [142-144]. The molecular signals that direct mitochondria to their cellular positions include: high local ADP:ATP in areas of high ATP demand [145], GTP hydrolysis by small GTP-binding proteins [146], actin-myosin-dependent nerve growth factor/TrkA/PI 3-kinase signaling [147], and various kinases [148,149]. Intrinsic mitochondrial proteins, such as OPA1, also may contribute to the selective distribution, clustering and stability of mitochondria in retinal neurons in addition to their role in mitochondrial fission and fusion, genome maintenance, and regulation of cristae morphology and substructure [150,151].

Eight new anatomical, ultrastructural and/or substructural differences between mouse rod spherules and cone pedicles were found. First, EM studies showed that there were three to five rows of rod spherules and a single row of larger, more electron lucent cone pedicles in the OPL. High magnification double labeling LSCM experiments, using either COX IV and M- and S-opsins or VDAC and rhodopsin, yielded complimentary results. Second, the cone pedicle volume is about 10 times larger than the rod spherule volume. Third, rod spherules contain a single very large ovoid mitochondrion that occupies 20-25% of the spherule volume, whereas cone pedicles contain an average of five medium-sized mitochondria that occupy 10-15% of the pedicle volume. Fourth, the rod spherule mitochondrion is located close to synaptic vesicle release sites, whereas cone pedicle mitochondria cluster in the distal part of the terminal. Fifth, the abundance of cristae membranes and correspondingly the cristae volume are two-fold greater in the rod spherule mitochondrion compared to an individual cone pedicle mitochondrion. Overall, however, the total amount of cristae membranes is 2 fold higher in cone pedicles than in rod spherules. Sixth, mitochondria CJ openings are 25% smaller in cone pedicle mitochondria than in rod spherule mitochondria. Seventh, networks of ER are in rod, as seen in rats [28], and cone synaptic terminals, although they are larger and denser in cone pedicles. Eighth, rod spherules uniformly and intensely stain for PMCA, whereas cone pedicles preferentially stain for NCX1 at their active zones. As reported by others, we found that mouse rod spherules mostly (about 95%) contain one ribbon synapse, but can contain two ribbon synaptic units [152,153], whereas cone pedicles contain 6-14 ribbon synapses [52,53; data not shown].

A comparison of our new substructural data on synaptic terminal mitochondria with that previously obtained on IS mitochondria [40,55], reveals three significant differences (see Figure 11). First, rod spherule and cone pedicle mitochondrial cristae segments (i.e., tubes and lamellae) do not exhibit high connectivity, whereas IS cristae segments are highly connected to each other so that a single crista can have many segments. CIS connectivity is maze-like [55]. Moreover, the number of segments per crista in CIS is significantly greater than in RIS. As opposed to IS mitochondria that connect many cristae segments together, rod spherule and cone pedicle mitochondria accommodate their greater amount of inner membrane by increasing the number of cristae. The mean number of cristae per mitochondrial volume for rod spherules and cone pedicles was about 1,300 and about 1,100, respectively, whereas it was about 570 and about 520 for RIS and CIS, respectively. Second, the cristae surface to mitochondrial surface ratio and the cristae volume to mitochondria volume ratio are 1.4- to 2.0 fold larger in CIS compared to RIS [55]. In marked contrast, the abundance of cristae membranes and correspondingly the cristae volume are 2.5 fold and 2.2 fold, respectively, greater in rod spherule mitochondria, than in each cone pedicle mitochondria. Third, the number of segments per crista were significantly greater (about 60%) in CIS compared to RIS, whereas it was similar and significantly lower in rod and cone synaptic terminal mitochondria. The reasons and mechanisms for these compartmental differences in cristae networking and crista formation are unknown. However, we interpret this data to indicate that mitochondrially mediated ATP production is greater in photoreceptor synaptic terminals than in the inner segments. This is consistent with findings that there is a strong Pasteur effect in the outer retina, such that glycolysis can support photoreceptor OS and IS function during mitochondrial inhibition [154].

The CJ opening in cone pedicle mitochondria is smaller than any previously measured in normal tissue or cells. Based on results that model the effects of matrix outward pressure on CJ diameter [155], we suggest that these differences in rod and cone mitochondria CJ diameter reflect small differences in matrix pressure. For example, when the matrix volume of mitochondria is experimentally increased, CJs become smaller and can pinch off leaving some cristae detached from the IBM [126,127]. The putative bioenergetic role of CJ remains to be determined. One hypothesis, consistent with the computer simulations, suggests that CJs partially regulate ATP generation by restricting the diffusion of ADP and ATP into and out of cristae [126]. That is, larger CJ openings facilitate and increase the capacity for ATP production. Alternatively, smaller CJs may restrict the diffusion of cytochrome c from the cristae lumen and thereby minimize diffusion distances that limit rates of electron transport needed for ATP synthesis. This would confer an energetic advantage to cone pedicle mitochondria and provide them with increased resistance to mitochondrially mediated apoptosis [40,156].

The biological and functional implications of the different number and position of mitochondria in rod spherules and cone pedicles are three-fold. The first relates to the optimal generation and utilization of ATP for various cellular process and synaptic transmission. The second involves the coordinated regulation of presynaptic Ca^2+^ levels necessary for synaptic transmission. The third is associated with potential Ca^2+^ overload and cell survival. The first consideration addresses two interrelated questions: (i) Is the ATP demand higher in a rod spherule or cone pedicle? (ii) Does a rod spherule or cone pedicle produce more ATP under normal physiological conditions? A number of observations strongly indicate that mouse (mammalian) cone pedicles have a higher ATP demand than rod spherules. Mouse cone pedicles have about 10 times more ribbons than rods (vide supra). Primate cone pedicles can have up to 50 synaptic ribbons [114,115]. Mammalian cone ribbons dock and tether approximately five-fold more vesicles than rod ribbons [116]. These vesicles require ATP for glutamate uptake and priming [33,34]. Cones have a faster initial component of exocytosis [18] and by extension a more rapid need to refill and prime the vesicle pool [34,117]. In addition, cones operate over a much larger dynamic range [118,119]. Biophysical and modeling experiments reveal that ATP demand logarithmically increases as the rate of luminance information transferred to second-order neurons increases [120], indicating that the metabolic cost of information transfer is higher in cones than rods. Photoreceptor NAKA, which has a specific activity three times higher than the whole retina [97,157] and consumes greater than 50% of the cell's ATP [158], is higher in cone pedicles than rod spherules [47]. One major reason for the higher Na^+^ pump activity and associated ATP demand in cone pedicles is that pedicles preferentially utilize NCX to extrude Ca^2+^ from their active zones (this paper; see schematic in Figure 14), and NCX activity is mostly controlled by NAKA activity [106]. In addition, selected areas of the cone axon and synaptic terminal utilize PMCA to extrude Ca^2+^, whereas this is the major Ca^2+^ extrusion mechanism in rod spherules (this paper). Moreover, our detailed results are different from those obtained in the calyx-type presynaptic nerve terminals of the chick ciliary ganglia [6,106], cultured mouse brain astrocytes [6,106] and rabbit skeletal muscle [107]. Interestingly, the presynaptic distribution of NCX and PMCA in mouse cone pedicles is opposite to that in the chick ciliary ganglia [106]. This difference may relate to the presence of N-type Ca^2+^ channels in the presynaptic terminal of the chick ganglia [159] as opposed to L-type Ca^2+^ channels in photoreceptor synapses [161,162].

ATP production in cone pedicle mitochondria is also likely greater than that in rod spherules. This is similar to our findings on RIS and CIS mitochondria [55,56]. Although the rod spherule mitochondrion has 2 fold more crista surface area than each cone mitochondrion, cone pedicles have five mitochondria per synaptic terminal and thus twice the total amount of cristae membranes. In addition, the CJ openings are smaller in cones, which could lead to more effective coupling of the electron transport chain to ATP synthesis [126]. Together, these should facilitate increased ATP production as evidenced by the higher COX activity in cone pedicles, compared to rod spherules [47]. Moreover, mammalian cone pedicles, but not rod spherules, contain glycogen and the phosphorylase isozyme necessary to convert it to glucose [162,163]. In contrast, the cone pedicle volume is large and the mitochondria cluster some distance from active zones. These factors will decrease the overall effective cellular ATP concentration at the active zones since the ATP diffusion distance is small [139]. Nevertheless, and likely more important for regulating the pedicle Ca^2+^ concentration, these clustered mitochondria will provide ATP for the nearby PMCA (see high magnification LSCM Figure 8D,E).

It is well known that strong metabolic coupling and cross-talk between mitochondria, ER, NCX, PMCA, and Ca^2+^ channels regulate presynaptic Ca^2+^ dynamics and neurotransmitter release [1,2,5,37,164]. Our high magnification immunocytochemical studies revealed several significant, and presumably functionally relevant, differences in the location and distribution of mitochondria, PMCA and NCX in rod spherules and cone pedicles. As noted, the rod spherule mitochondrion is located close to synaptic vesicle release sites, whereas cone pedicle mitochondria cluster in the distal part of the terminal. Neuronal mitochondria have a low affinity, but high capacity for Ca^2+^ (Kd approximately 1-5 μM; 1-5 μmol mg protein^-1^) [165,166]. NCX and PMCA are the two major Ca^2+^ transporters in the presynaptic terminal [2,106]. We found that in the cone axon the active zone of pedicles preferentially and intensely stained for NCX, whereas rod spherules diffusely stained for NCX. In contrast, rod spherules uniformly and intensely stained for PMCA, whereas only the distal portion of the cone pedicle stained for PMCA. Triple labeling experiments with PMCA, VDAC and either VGluT1 or PNA revealed that the cone pedicle mitochondria were in close proximity to the PMCA labeled membranes. NCX utilizes the activity and gradient established by NAKA to maintain low intracellular Ca^2+^ levels, and is spatiotemporally associated with the two high ouabain affinity isozymes in neurons (α2: NAKA2 or α3: NAKA3). Immunocytochemical and biochemical experiments show that NAKA3 is present in rodent IS and synaptic terminals [97,167]. NCX has a low affinity and high turnover rate for Ca^2+^ extrusion (Kd approximately 1 μM; kcat 2000-5000 s^-1^), whereas PMCA has a high affinity and a low turnover rate for Ca^2+^ extrusion (Kd approximately 0.1 μM; kcat approximately 30-250 s^-1^) [106,168]. This suggests that the synaptic terminal Ca^2+^ concentration is rapidly lowered by NCX to its Kd value, while the high affinity PMCA slowly lowers it to a dark-adapted value of 0.3-2 μM [19,26]. ER cisternae were widely distributed throughout the rod and cone terminals and often were in close apposition to mitochondria, as observed in other tissues [89,104,105]. SERCA3 extensively labeled the IS and OPL. It has a low affinity and relatively low turnover rate (Kd approximately 1-2 μM; kcat approximately 130-150 s^-1^), but ER have a high capacity for Ca^2+^ uptake (0.5-1 μM) [169,170]. This is consistent with the role of ER Ca^2+^ uptake, which helps lower the intraterminal Ca^2+^ concentration with PMCA and NCX.

Our results, in concert with those from many other studies, enabled us to construct a spatiotemporal model of rod spherule and cone pedicle regulation of Ca^2+^ levels during darkness (see Figure 14 for illustration) and light adaptation. In the dark-adapted mouse retina, a low level of Ca^2+^ enters rod and cone synaptic terminals through Ca_v_1.4 and Ca_v_1.3 channels, respectively [160,161], and glutamate is continuously released. Although dark-adapted mammalian rods are slightly more depolarized than cones [61], it appears that the activation values for these voltage-gated calcium channels match their resting membrane potentials [161,171]. This should produce one or more, depending upon the number of active zones, overlapping microdomains of elevated plasmalemmal Ca^2+^. By extension, this should result in higher Ca^2+^ concentrations around the cone active zones, compared to the rod active zone, which likely accounts for the 10 fold faster initial rate of exocytosis in salamander cones compared to rods [18]. In dark-adapted salamander rod terminals, the intraterminal Ca^2+^ concentration is estimated to range from 0.3-2 μM [19,26], whereas the microdomains likely contain 20-40 μM Ca^2+^ [17,172]. Some of this Ca^2+^ in the microdomain will bind to CaBP4, a Ca^2+^ binding protein in photoreceptor terminals that directly associates with Ca_v_1.4 channels [173], and synaptotagmin 1 in the active zone [70,113]. Following light adaptation, NCX should rapidly remove much of the remaining intraterminal Ca^2+^ in cooperation with the high capacity ER (SERCA3-positive): especially in cone pedicles where NCX localizes to the active zones. However, NCX will only lower the intraterminal Ca^2+^ until the ATP-dependent NAKA3 re-establishes the Na^+^ gradient. In cones, further Ca^2+^ extrusion must be facilitated by the slower ATP-dependent PMCAs, which can maintain submicromolar levels of Ca^2+^ [174,175]. Presumably, in rods the PMCAs are working continuously to maintain a spatially averaged Ca^2+^ concentration of 0.3-2 μM: the concentration estimated to maintain vesicle cycling in the salamander rod [19,26].

To test this spatiotemporal model of [Ca^2+^] regulation in dark- and light-adapted rod spherules and cone pedicles, we conducted Ca^2+^ imaging studies on retinal slices utilizing fluo-3: a high affinity fluorescent Ca^2+^ dye. These experiments produced three novel and functionally relevant results that validated several of our predictions. First, in dark-adapted retinas the estimated free [Ca^2+^] in rod spherules was about 2 μM, which was significantly (3.2 fold) lower than that in cones pedicles. These findings are consistent with the interpretation of our detailed immunocytochemical findings. That is, rod spherules predominantly possess high affinity/low turnover PMCA that maintains a low intraterminal [Ca^2+^] in order to increase the sensitivity and signal-to-noise ratio of rods [17-21]. Furthermore, the RFI appeared higher in the presumed location of the rod spherule mitochondria than in the cone pedicle area where mitochondria cluster. This observation is in agreement with our suggestion that rod spherule mitochondria actively participate in intraterminal Ca^2+^ buffering, which also increases their ATP production capacity. The recent findings of Krizaj and colleagues [176], who compared the morphology and light responses of retinas from control mice and deafwaddler dfw2J mice that lack the functional PMCA2 protein, confirm our suggestion and results that PMCA-mediated Ca^2+^ extrusion selectively modulates the rod spherules and not the cone pedicles. That is, the rod-mediated electroretinogram b-waves recorded from dfw2J mice were markedly smaller and slower than those in control mice, however, there were no alterations in the cone-mediated b-wave. At the light microscopy level, the retinas from both mice looked similar.

Second, during light adaptation the estimated free [Ca^2+^] in cone pedicles was 0.23 μM, which is significantly (3.2 fold) lower than that in rod spherules. This is consistent with our immunocytochemical, EM and ET findings that cone pedicles possess the structural components and functional mechanisms (e.g., low affinity/high turnover NCX) to more rapidly remove intraterminal free Ca^2+^ compared to rod spherules as well as PMCA to lower the free [Ca^2+^] below the Kd of NCX. Furthermore, it is compatible with the observation that the recovery and kinetics of exocytosis is faster in cones than rods [9,18,118,119].

Third, in dark-adapted retinas Ca^2+^ microdomains were observed adjacent to the plasmalemma and synaptic ribbons of rod spherules and cone pedicles. These Ca^2+^ microdomains were larger in cone pedicles than rod spherules and they overlapped in cone pedicles. Interestingly, the estimated size of the Ca^2+^ microdomains in dark-adapted cone pedicles were similar to those measured in dark-adapted goldfish Mb1 retinal cells [24]. Although the relation between Ca^2+^ microdomains in rod and cone synaptic terminals and the regulation of exocytosis remains to be determined, our Ca^2+^ imaging findings suggest that cone pedicles will release more synaptic vesicles and exhibit faster exocytosis than rods spherules. This is consistent with the physiology of these two photoreceptor terminals [9,17-21,36] and the goldfish Mb1 retinal cells [9,24,36]. Ca^2+^ microdomains, which cluster near Ca^2+^ channels, were visualized at the ribbon synapses of frog saccular hair cells, turtle cochlear hair cells and goldfish retinal Mb1 cells [24,177-179].

The mitochondria certainly provide the Mg-ATP necessary for NAKA3, SERCA3 and PMCA in both rod spherules and cone pedicles. The proximity of the rod spherule mitochondria to the active zone and its relatively high Ca^2+^ concentration (at least 20 μM) leads us to speculate that these mitochondria play two additional important and interrelated roles. First, they may participate in local Ca^2+^ buffering in order to limit the expansion of the Ca^2+^ microdomain [24] and thereby maintain the rod's single photon sensitivity [20,21]. This likely operates because as the cytosolic ATP concentration decreases and ADP concentration increases, the electrogenic mitochondrial Ca^2+^ carrier (uniporter) increases its rate of Ca^2+^ uptake and retention of Ca^2+^ loads. When the cytosolic Ca^2+^ concentration returns to baseline, mitochondria release their stored Ca^2+^ loads [165,166]. Second, the activity of the mitochondrial dehydrogenases increases in response to the increase in the free mitochondrial matrix Ca^2+^ concentration. This results in an increase in the rate of electron transport and subsequently the amount of ATP production [180,181]. In summary, we suggest that reciprocal cooperation between mitochondria, ER and the Ca^2+^ transporters in rod spherules and cone pedicles during darkness control presynaptic Ca^2+^ handling, ATP production and glutamate release (Figure 14).

The third possibility, though not mutually exclusive from the first two, is that cone pedicle mitochondria cluster at a distance from active sites to protect themselves from Ca^2+^ overload during synaptic transmitter release. As noted, in dark-adapted retinas there are likely numerous overlapping microdomains of elevated Ca^2+^ close to the synaptic ribbons of the cone pedicle. Moreover, this steady Ca^2+^ influx could produce a Ca^2+^ induced Ca^2+^ release (CICR) from presynaptic rod and cone SER [28; this paper], which would preferentially produce Ca^2+^ overload in cone pedicle mitochondria. In the salamander rod synapse, there is a ryanodine-dependent amplification mechanism that couples CICR with continuous vesicle release [26,27]. Consistent with this concept, a recent report demonstrated that synaptic mitochondria are more susceptible to Ca^2+^ overload and mitochondrial permeability transition than non-synaptic mitochondria [182]. In addition, a similar phenomenon may occur in rod and cone synaptic terminals, like in RIS [40,94], as rod spherule mitochondria undergo permeability transition following postnatal only developmental lead exposure [data not shown, manuscript in preparation]. Moreover, both rod and cone synaptic mitochondria undergo permeability transition in an HRG4 mutant mice that models human cone-rod dystrophy [183].

In summary, rod spherules and cone pedicles have significantly different morphology, ultrastructure, mitochondrial density and location, patterns of protein expression, and regulatory mechanisms that control their [Ca^2+^] during dark and light-adaptation. Compared to rod spherule mitochondria, individual cone pedicle mitochondria are smaller and more ovoid, reside more remotely from the active zone, possess less cristae surface and volume, and have a very small CJ diameter. These distinct characteristics play a fundamental role in the ability of rod and cone presynaptic terminals to regulate and produce ATP as well as to buffer Ca^2+^ microdomains. In addition, the ER, PMCA and NCX all play essential and inter-dependent roles in rod and cone presynaptic terminals. Their differences in cellular distribution and location, amount of protein expression, affinity and capacity for Ca^2+^ and their turnover rate of Ca^2+^ significantly contribute to the kinetics of exocytosis in rods and cones. The results from these LSCM, EM, ET, and Ca^2+^ imaging experiments can help design new electrophysiological, pharmacological, molecular/transgenic and imaging experiments that will enable us to gain a deeper and fuller understanding of the spatiotemporal control of vertebrate rod and cone synaptic terminal Ca^2+^ concentrations and mitochondrial ATP production as they relate to exocytosis and endocytosis. In addition, our results illustrate the need to identify and determine the spatial distribution, compartment-specific differences and interrelation of Ca^2+^-handling proteins and mitochondria in other neural tissues so that the structure-function relations in different neurons and chemical synapses can be ascertained. Moreover, additional new retinal experiments will provide important data on the role and sensitivity of rod and cone synaptic terminal mitochondria to Ca^2+^ overload and cell injury as compared to RIS and CIS mitochondria [39,40,182]. Finally, we hope that the results from our work, the suggested experiments as well as future experiments will provide further groundwork for understanding, and treating, the pathophysiological effects and visual deficits that result from inherited, disease-related as well as chemically- and pharmacologically-induced retinal degenerations.

## References

[1] Augustine GJ How does calcium trigger neurotransmitter release? Curr Opin Neurobiol 2001 11 320 6 11399430 10.1016/s0959-4388(00)00214-2

[2] Berridge MJ Bootman MD Roderick HL Calcium signalling: dynamics, homeostasis and remodelling. Nat Rev Mol Cell Biol 2003 4 517 29 12838335 10.1038/nrm1155

[3] Augustine GJ Burns ME DeBello WM Pettit DL Schweizer FE Exocytosis: proteins and perturbations. Annu Rev Pharmacol Toxicol 1996 36 659 701 8725405 10.1146/annurev.pa.36.040196.003303

[4] Li L Chin LS The molecular machinery of synaptic vesicle exocytosis. Cell Mol Life Sci 2003 60 942 60 12827282 10.1007/s00018-003-2240-7 PMC11138869

[5] Guerini D Coletto L Carafoli E Exporting calcium from cells. Cell Calcium 2005 38 281 9 16102821 10.1016/j.ceca.2005.06.032

[6] Juhaszova M Church P Blaustein MP Stanley EF Location of calcium transporters at presynaptic terminals. Eur J Neurosci 2000 12 839 46 10762313 10.1046/j.1460-9568.2000.00974.x

[7] Billups B Forsythe ID Presynaptic mitochondrial calcium sequestration influences transmission at mammalian central synapses. J Neurosci 2002 22 5840 7 12122046 10.1523/JNEUROSCI.22-14-05840.2002 PMC6757942

[8] David G Barrett EF Mitochondrial Ca^2+^ uptake prevents desynchronization of quantal release and minimizes depletion during repetitive stimulation of mouse motor nerve terminals. J Physiol 2003 548 425 38 12588898 10.1113/jphysiol.2002.035196 PMC2342850

[9] Heidelberger R Thoreson WB Witkovsky P Synaptic transmission at retinal ribbon synapses. Prog Retin Eye Res 2005 24 682 720 16027025 10.1016/j.preteyeres.2005.04.002 PMC1383430

[10] Ohnuma K Kazawa T Ogawa S Suzuki N Miwa A Kijima H Cooperative Ca^2+^ removal from presynaptic terminals of the spiny lobster neuromuscular junction. Biophys J 1999 76 1819 34 10096881 10.1016/S0006-3495(99)77342-X PMC1300159

[11] Hongpaisan J Pivovarova NB Colegrove SL Leapman RD Friel DD Andrews SB Multiple modes of calcium-induced calcium release in sympathetic neurons II: a [Ca^2+^](i)- and location-dependent transition from endoplasmic reticulum Ca accumulation to net Ca release. J Gen Physiol 2001 118 101 12 11429447 10.1085/jgp.118.1.101 PMC2233743

[12] Kobayashi K Tachibana M Ca^2+^ regulation in the presynaptic terminals of goldfish retinal bipolar cells. J Physiol 1995 483 79 94 7539842 10.1113/jphysiol.1995.sp020569 PMC1157873

[13] von Gersdorff H Vardi E Matthews G Sterling P Evidence that vesicles on the synaptic ribbon of retinal bipolar neurons can be rapidly released. Neuron 1996 16 1221 7 8663998 10.1016/s0896-6273(00)80148-8

[14] Zenisek D Matthews G The role of mitochondria in presynaptic calcium handling at a ribbon synapse. Neuron 2000 25 229 37 10707986 10.1016/s0896-6273(00)80885-5

[15] Oikawa T Ogawa T Motokawa K Origin of so-called cone action potential. J Neurophysiol 1959 22 102 11 13621260 10.1152/jn.1959.22.1.102

[16] Toyoda J Hashimoto H Anno H Tomita T The rod response in the frog and studies by intracellular recording. Vision Res 1970 10 1093 100 4101507 10.1016/0042-6989(70)90026-x

[17] Thoreson WB Rabl K Townes-Anderson E Heidelberger R A highly Ca^2+^-sensitive pool of vesicles contributes to linearity at the rod photoreceptor ribbon synapse. Neuron 2004 42 595 605 15157421 10.1016/s0896-6273(04)00254-5 PMC3108437

[18] Rabl K Cadetti L Thoreson WB Kinetics of exocytosis is faster in cones than in rods. J Neurosci 2005 25 4633 40 Erratum in: J Neurosci. 2005; 25:table of contents 15872111 10.1523/JNEUROSCI.4298-04.2005 PMC1383432

[19] Rieke F Schwartz EA Asynchronous transmitter release: control of exocytosis and endocytosis at the salamander rod synapse. J Physiol 1996 493 1 8 8735690 10.1113/jphysiol.1996.sp021360 PMC1158946

[20] Baylor DA Lamb TD Yau KW Responses of retinal rods to single photons. J Physiol 1979 288 613 34 112243 PMC1281447

[21] Field GD Rieke F Nonlinear signal transfer from mouse rods to bipolar cells and implications for visual sensitivity. Neuron 2002 34 773 85 12062023 10.1016/s0896-6273(02)00700-6

[22] Murakami M Otsuka T Shimazaki H Effects of aspartate and glutamate on the bipolar cells in the carp retina. Vision Res 1975 15 456 8 166508 10.1016/0042-6989(75)90101-7

[23] Miller AM Schwartz EA Evidence for the identification of synaptic transmitters released by photoreceptors of the toad retina. J Physiol 1983 334 325 49 6134824 10.1113/jphysiol.1983.sp014497 PMC1197317

[24] Beaumont V Llobet A Lagnado L Expansion of calcium microdomains regulates fast exocytosis at a ribbon synapse. Proc Natl Acad Sci USA 2005 102 10700 5 16027365 10.1073/pnas.0501961102 PMC1180766

[25] Morgans CW El Far O Berntson A Wassle H Taylor WR Calcium extrusion from mammalian photoreceptor terminals. J Neurosci 1998 18 2467 74 9502807 10.1523/JNEUROSCI.18-07-02467.1998 PMC6793104

[26] Krizaj D Bao JX Schmitz Y Witkovsky P Copenhagen DR Caffeine-sensitive calcium stores regulate synaptic transmission from retinal rod photoreceptors. J Neurosci 1999 19 7249 61 10460231 10.1523/JNEUROSCI.19-17-07249.1999 PMC6782489

[27] Suryanarayanan A Slaughter MM Synaptic transmission mediated by internal calcium stores in rod photoreceptors. J Neurosci 2006 26 1759 66 16467524 10.1523/JNEUROSCI.3895-05.2006 PMC6793629

[28] Ladman AJ The fine structure of the rod-bipolar cell synapse in the retina of the albino rat. J Biophys Biochem Cytol 1958 4 459 66 13563552 10.1083/jcb.4.4.459 PMC2224491

[29] Mercurio AM Holtzman E Smooth endoplasmic reticulum and other agranular reticulum in frog retinal photoreceptors. J Neurocytol 1982 11 263 93 6978386 10.1007/BF01258247

[30] Ungar F Piscopo I Letizia J Holtzman E Uptake of calcium by the endoplasmic reticulum of the frog photoreceptor. J Cell Biol 1984 98 1645 55 6609924 10.1083/jcb.98.5.1645 PMC2113183

[31] Krizaj D Serca isoform expression in the mammalian retina. Exp Eye Res 2005 81 690 9 15967430 10.1016/j.exer.2005.04.007 PMC2921800

[32] Krizaj D Demarco SJ Johnson J Strehler EE Copenhagen DR Cell-specific expression of plasma membrane calcium ATPase isoforms in retinal neurons. J Comp Neurol 2002 451 1 21 12209837 10.1002/cne.10281 PMC1987379

[33] Ozkan ED Ueda T Glutamate transport and storage in synaptic vesicles. Jpn J Pharmacol 1998 77 1 10 9639055 10.1254/jjp.77.1

[34] Heidelberger R Sterling P Matthews G Roles of ATP in depletion and replenishment of the releasable pool of synaptic vesicles. J Neurophysiol 2002 88 98 106 12091535 10.1152/jn.2002.88.1.98

[35] Dowling JE Boycott BB Organization of the primate retina: electron microscopy. Proc R Soc Lond B Biol Sci 1966 166 80 111 4382694 10.1098/rspb.1966.0086

[36] Sterling P Matthews G Structure and function of ribbon synapses. Trends Neurosci 2005 28 20 9 15626493 10.1016/j.tins.2004.11.009

[37] Barrett EF Contrasting contributions of endoplasmic reticulum and mitochondria to Ca(2)+ handling in neurons. J Gen Physiol 2001 118 79 82 11429445 10.1085/jgp.118.1.79 PMC2233750

[38] Heidelberger R Adenosine triphosphate and the late steps in calcium-dependent exocytosis at a ribbon synapse. J Gen Physiol 1998 111 225 41 9450941 10.1085/jgp.111.2.225 PMC2222770

[39] Fox DA Poblenz AT He L Calcium overload triggers rod photoreceptor apoptotic cell death in chemical-induced and inherited retinal degenerations. Ann N Y Acad Sci 1999 893 282 5 10672249 10.1111/j.1749-6632.1999.tb07837.x

[40] He L Perkins GA Poblenz AT Harris JB Hung M Ellisman MH Fox DA Bcl-x_L_ overexpression blocks bax-mediated mitochondrial contact site formation and apoptosis in rod photoreceptors of lead-exposed mice. Proc Natl Acad Sci USA 2003 100 1022 7 12540825 10.1073/pnas.0333594100 PMC298719

[41] Erecinska M Silver IA ATP and brain function. J Cereb Blood Flow Metab 1989 9 2 19 2642915 10.1038/jcbfm.1989.2

[42] Smith RA Ord MJ Mitochondrial form and function relationships in vivo: their potential in toxicology and pathology. Int Rev Cytol 1983 83 63 134 6196312 10.1016/s0074-7696(08)61686-1

[43] Kadenbach B Barth J Akgun R Freund R Linder D Possekel S Regulation of mitochondrial energy generation in health and disease. Biochim Biophys Acta 1995 1271 103 9 7599196 10.1016/0925-4439(95)00016-w

[44] Shepherd GM Harris KM Three-dimensional structure and composition of CA3-->CA1 axons in rat hippocampal slices: implications for presynaptic connectivity and compartmentalization. J Neurosci 1998 18 8300 10 9763474 10.1523/JNEUROSCI.18-20-08300.1998 PMC6792846

[45] Graymore CN. General aspects of metabolism in the retina. In: The Eye. Davson H, editor. Vol 1. 2nd ed. New York: Academic Press, 1969. p. 601-45.

[46] Cohen AI The fine structure of the extrafoveal receptors of the Rhesus monkey. Exp Eye Res 1961 1 128 36 13880203 10.1016/s0014-4835(61)80018-3

[47] Kageyama GH Wong-Riley MT The histochemical localization of cytochrome oxidase in the retina and lateral geniculate nucleus of the ferret, cat, and monkey, with particular reference to retinal mosaics and ON/OFF-center visual channels. J Neurosci 1984 4 2445 59 6092560 10.1523/JNEUROSCI.04-10-02445.1984 PMC6564714

[48] Chen E Soderberg PG Lindstrom B Activity distribution of cytochrome oxidase in the rat retina. A quantitative histochemical study. Acta Ophthalmol (Copenh) 1989 67 645 51 2559572 10.1111/j.1755-3768.1989.tb04396.x

[49] Medrano CJ Fox DA Oxygen consumption in the rat outer and inner retina: light- and pharmacologically-induced inhibition. Exp Eye Res 1995 61 273 84 7556491 10.1016/s0014-4835(05)80122-8

[50] Linsenmeier RA Effects of light and darkness on oxygen distribution and consumption in the cat retina. J Gen Physiol 1986 88 521 42 3783124 10.1085/jgp.88.4.521 PMC2228847

[51] Hoang QV Linsenmeier RA Chung CK Curcio CA Photoreceptor inner segments in monkey and human retina: mitochondrial density, optics, and regional variation. Vis Neurosci 2002 19 395 407 12511073 10.1017/s0952523802194028

[52] Olney JW An electron microscopic study of synapse formation, receptor outer segment development, and other aspects of developing mouse retina. Invest Ophthalmol 1968 7 250 68 5655873

[53] Carter-Dawson LD LaVail MM Rods and cones in the mouse retina. I. Structural analysis using light and electron microscopy. J Comp Neurol 1979 188 245 62 500858 10.1002/cne.901880204

[54] Smith RG Freed MA Sterling P Microcircuitry of the dark-adapted cat retina: functional architecture of the rod-cone network. J Neurosci 1986 6 3505 17 3794785 10.1523/JNEUROSCI.06-12-03505.1986 PMC6568666

[55] Perkins GA Ellisman MH Fox DA Three-dimensional analysis of mouse rod and cone mitochondrial cristae architecture: bioenergetic and functional implications. Mol Vis 2003 9 60 73 http://www.molvis.org/molvis/v9/a10/12632036

[56] Perkins GA Ellisman MH Fox DA The structure-function correlates of mammalian rod and cone photoreceptor mitochondria: observations and unanswered questions. Mitochondrion 2004 4 695 703 16120425 10.1016/j.mito.2004.07.020

[57] Kolb H Anatomical pathways for color vision in the human retina. Vis Neurosci 1991 7 61 74 1931801 10.1017/s0952523800010944

[58] Blanks JC Johnson LV Specific binding of peanut lectin to a class of retinal photoreceptor cells. A species comparison. Invest Ophthalmol Vis Sci 1984 25 546 57 6715128

[59] Korenbrot JI Ca^2+^ flux in retinal rod and cone outer segments: differences in Ca^2+^ selectivity of the cGMP-gated ion channels and Ca^2+^ clearance rates. Cell Calcium 1995 18 285 300 8556768 10.1016/0143-4160(95)90025-x

[60] Picones A Korenbrot JI Permeability and interaction of Ca^2+^ with cGMP-gated ion channels differ in retinal rod and cone photoreceptors. Biophys J 1995 69 120 7 7545443 10.1016/S0006-3495(95)79881-2 PMC1236230

[61] Schneeweis DM Schnapf JL Photovoltage of rods and cones in the macaque retina. Science 1995 268 1053 6 7754386 10.1126/science.7754386

[62] Wong-Riley MT Huang Z Liebl W Nie F Xu H Zhang C Neurochemical organization of the macaque retina: effect of TTX on levels and gene expression of cytochrome oxidase and nitric oxide synthase and on the immunoreactivity of Na^+^ K^+^ ATPase and NMDA receptor subunit I. Vision Res 1998 38 1455 77 9667011 10.1016/s0042-6989(98)00001-7

[63] Scarpelli DG Craig EL The fine localization of nucleoside triphosphatase activity in the retina of the frog. J Cell Biol 1963 17 279 88 13976534 10.1083/jcb.17.2.279 PMC2106202

[64] Fox DA Chu LW Rods are selectively altered by lead: II. Ultrastructure and quantitative histology. Exp Eye Res 1988 46 613 25 2838312 10.1016/s0014-4835(88)80017-4

[65] Zhu X Brown B Li A Mears AJ Swaroop A Craft CM GRK1-dependent phosphorylation of S and M opsins and their binding to cone arrestin during cone phototransduction in the mouse retina. J Neurosci 2003 23 6152 60 12853434 10.1523/JNEUROSCI.23-14-06152.2003 PMC6740345

[66] Szel A Rohlich P Caffe AR van Veen T Distribution of cone photoreceptors in the mammalian retina. Microsc Res Tech 1996 35 445 62 9016448 10.1002/(SICI)1097-0029(19961215)35:6<445::AID-JEMT4>3.0.CO;2-H

[67] Haverkamp S Ghosh KK Hirano AA Wassle H Immunocytochemical description of five bipolar cell types of the mouse retina. J Comp Neurol 2003 455 463 76 12508320 10.1002/cne.10491 PMC2834891

[68] Haverkamp S Wassle H Immunocytochemical analysis of the mouse retina. J Comp Neurol 2000 424 1 23 10888735

[69] Sherry DM Wang MM Bates J Frishman LJ Expression of vesicular glutamate transporter 1 in the mouse retina reveals temporal ordering in development of rod vs. cone and ON vs. OFF circuits. J Comp Neurol 2003 465 480 98 12975811 10.1002/cne.10838

[70] Heidelberger R Wang MM Sherry DM Differential distribution of synaptotagmin immunoreactivity among synapses in the goldfish, salamander, and mouse retina. Vis Neurosci 2003 20 37 49 12699082 10.1017/s095252380320105x

[71] Muresan V Lyass A Schnapp BJ The kinesin motor KIF3A is a component of the presynaptic ribbon in vertebrate photoreceptors. J Neurosci 1999 19 1027 37 9920666 10.1523/JNEUROSCI.19-03-01027.1999 PMC6782153

[72] Molday RS MacKenzie D Monoclonal antibodies to rhodopsin: characterization, cross-reactivity, and application as structural probes. Biochemistry 1983 22 653 60 6188482 10.1021/bi00272a020

[73] Taanman JW Hall RE Tang C Marusich MF Kennaway NG Capaldi RA Tissue distribution of cytochrome c oxidase isoforms in mammals. Characterization with monoclonal and polyclonal antibodies. Biochim Biophys Acta 1993 1225 95 100 8241294 10.1016/0925-4439(93)90128-n

[74] McEnery MW Dawson TM Verma A Gurley D Colombini M Snyder SH Mitochondrial voltage-dependent anion channel. Immunochemical and immunohistochemical characterization in rat brain. J Biol Chem 1993 268 23289 96 8226852

[75] Winkelbach H Walter G Morys-Wortmann C Paetzold G Hesse D Zimmerman B Florke H Reyman S Stadtmuller U Thinnes FP Hilschmann N Studies on human porin. XII. Eight monoclonal mouse anti-"porin 31HL" antibodies discriminate type 1 and type 2 mammalian porin channels/VDACs in western blotting and enzyme-linked immunosorbent assays. Biochem Med Metab Biol 1994 52 120 7 7993658 10.1006/bmmb.1994.1042

[76] Ropp PA Copeland WC Cloning and characterization of the human mitochondrial DNA polymerase, DNA polymerase gamma. Genomics 1996 36 449 58 8884268 10.1006/geno.1996.0490

[77] Davis AF Ropp PA Clayton DA Copeland WC Mitochondrial DNA polymerase gamma is expressed and translated in the absence of mitochondrial DNA maintenance and replication. Nucleic Acids Res 1996 24 2753 9 8759007 10.1093/nar/24.14.2753 PMC146014

[78] Corbett EF Michalak M Calcium, a signaling molecule in the endoplasmic reticulum? Trends Biochem Sci 2000 25 307 11 10871879 10.1016/s0968-0004(00)01588-7

[79] Camacho P Lechleiter JD Calreticulin inhibits repetitive intracellular Ca^2+^ waves. Cell 1995 82 765 71 7671304 10.1016/0092-8674(95)90473-5

[80] Martin V Bredoux R Corvazier E Van Gorp R Kovacs T Gelebart P Enouf J Three novel sarco/endoplasmic reticulum Ca^2+^-ATPase (SERCA) 3 isoforms. Expression, regulation, and function of the membranes of the SERCA3 family. J Biol Chem 2002 277 24442 52 11956212 10.1074/jbc.M202011200

[81] Magyar CE White KE Rojas R Apodaca G Friedman PA Plasma membrane Ca^2+^-ATPase and NCX1 Na^+^/Ca^2+^ exchanger expression in distal convoluted tubule cells. Am J Physiol Renal Physiol 2002 283 F29 40 12060584 10.1152/ajprenal.00252.2000

[82] Philipson KD Longoni S Ward R Purification of the cardiac Na^+^-Ca^2+^ exchange protein. Biochim Biophys Acta 1988 945 298 306 3191125 10.1016/0005-2736(88)90492-0

[83] Cohen AI The ultrastructure of the rods of the mouse retina. Am J Anat 1960 107 23 48 13694328 10.1002/aja.1001070103

[84] Leure-Dupree AE Observations on the synaptic organization of the retina of the albino rat: a light and electron microscopic study. J Comp Neurol 1974 153 149 78 4810723 10.1002/cne.901530203

[85] Medrano CJ Fox DA Substrate-dependent effects of calcium on rat retinal mitochondrial respiration: physiological and toxicological studies. Toxicol Appl Pharmacol 1994 125 309 21 8171438 10.1006/taap.1994.1077

[86] Szel A Rohlich P Caffe AR Juliusson B Aguirre G Van Veen T Unique topographic separation of two spectral classes of cones in the mouse retina. J Comp Neurol 1992 325 327 42 1447405 10.1002/cne.903250302

[87] Jacobs GH Fenwick JA Williams GA Cone-based vision of rats for ultraviolet and visible lights. J Exp Biol 2001 204 2439 46 11511659 10.1242/jeb.204.14.2439

[88] Kueng-Hitz N Grimm C Lansel N Hafezi F He L Fox DA Reme CE Niemeyer G Wenzel A The retina of c-fos-/- mice: electrophysiologic, morphologic and biochemical aspects. Invest Ophthalmol Vis Sci 2000 41 909 16 10711713

[89] Perkins G Renken C Martone ME Young SJ Ellisman M Frey T Electron tomography of neuronal mitochondria: three-dimensional structure and organization of cristae and membrane contacts. J Struct Biol 1997 119 260 72 9245766 10.1006/jsbi.1997.3885

[90] Perkins GA Renken CW Song JY Frey TG Young SJ Lamont S Martone ME Lindsey S Ellisman MH Electron tomography of large, multicomponent biological structures. J Struct Biol 1997 120 219 27 9441927 10.1006/jsbi.1997.3920

[91] Mastronarde DN Dual-axis tomography: an approach with alignment methods that preserve resolution. J Struct Biol 1997 120 343 52 9441937 10.1006/jsbi.1997.3919

[92] Lawrence A Bouwer JC Perkins G Ellisman MH Transform-based backprojection for volume reconstruction of large format electron microscope tilt series. J Struct Biol 2006 154 144 67 16542854 10.1016/j.jsb.2005.12.012

[93] Perkins GA Renken CW Frey TG Ellisman MH Membrane architecture of mitochondria in neurons of the central nervous system. J Neurosci Res 2001 66 857 65 11746412 10.1002/jnr.10050

[94] He L Poblenz AT Medrano CJ Fox DA Lead and calcium produce rod photoreceptor cell apoptosis by opening the mitochondrial permeability transition pore. J Biol Chem 2000 275 12175 84 10766853 10.1074/jbc.275.16.12175

[95] Werblin FS Transmission along and between rods in the tiger salamander retina. J Physiol 1978 280 449 70 211229 10.1113/jphysiol.1978.sp012394 PMC1282669

[96] Williams DA, Cody SH, Dubbin PN. Introducing and calibrating fluorescent probes in cells and organelles. In: Mason WT, editor. Fluorescent and Luminescent Probes for Biological Activity. New York: Academic Press; 1993. p. 321-34.

[97] Dyatlov VA Dyatlova OM Parsons PJ Lawrence DA Carpenter DO Lipopolysaccharide and interleukin-6 enhance lead entry into cerebellar neurons: application of a new and sensitive flow cytometric technique to measure intracellular lead and calcium concentrations. Neurotoxicology 1998 19 293 302 9553966

[98] Minta A Kao JP Tsien RY Fluorescent indicators for cytosolic calcium based on rhodamine and fluorescein chromophores. J Biol Chem 1989 264 8171 8 2498308

[99] Kao JP Harootunian AT Tsien RY Photochemically generated cytosolic calcium pulses and their detection by fluo-3. J Biol Chem 1989 264 8179 84 2498309

[100] Perez-Terzic C Stehno-Bittel L Clapham DE Nucleoplasmic and cytoplasmic differences in the fluorescence properties of the calcium indicator Fluo-3. Cell Calcium 1997 21 275 82 9160163 10.1016/s0143-4160(97)90115-9

[101] Shulman LM Fox DA Dopamine inhibits mammalian photoreceptor Na^+^,K^+^-ATPase activity via a selective effect on the alpha3 isozyme. Proc Natl Acad Sci USA 1996 93 8034 9 8755598 10.1073/pnas.93.15.8034 PMC38870

[102] Lumpkin EA Hudspeth AJ Regulation of free Ca^2+^ concentration in hair-cell stereocilia. J Neurosci 1998 18 6300 18 9698322 10.1523/JNEUROSCI.18-16-06300.1998 PMC6793210

[103] Burmeister M Novak J Liang MY Basu S Ploder L Hawes NL Vidgen D Hoover F Goldman D Kalnins VI Roderick TH Taylor BA Hankin MH McInnes RR Ocular retardation mouse caused by Chx10 homeobox null allele: impaired retinal progenitor proliferation and bipolar cell differentiation. Nat Genet 1996 12 376 84 8630490 10.1038/ng0496-376

[104] Rutter GA Rizzuto R Regulation of mitochondrial metabolism by ER Ca^2+^ release: an intimate connection. Trends Biochem Sci 2000 25 215 21 10782088 10.1016/s0968-0004(00)01585-1

[105] Vandecasteele G Szabadkai G Rizzuto R Mitochondrial calcium homeostasis: mechanisms and molecules. IUBMB Life 2001 52 213 9 11798035 10.1080/15216540152846028

[106] Blaustein MP Juhaszova M Golovina VA Church PJ Stanley EF Na/Ca exchanger and PMCA localization in neurons and astrocytes: functional implications. Ann N Y Acad Sci 2002 976 356 66 12502582 10.1111/j.1749-6632.2002.tb04762.x

[107] Sacchetto R Margreth A Pelosi M Carafoli E Colocalization of the dihydropyridine receptor, the plasma-membrane calcium ATPase isoform 1 and the sodium/calcium exchanger to the junctional-membrane domain of transverse tubules of rabbit skeletal muscle. Eur J Biochem 1996 237 483 8 8647089 10.1111/j.1432-1033.1996.0483k.x

[108] Ketelaars SO Gorter JA Aronica E Wadman WJ Calcium extrusion protein expression in the hippocampal formation of chronic epileptic rats after kainate-induced status epilepticus. Epilepsia 2004 45 1189 201 15461673 10.1111/j.0013-9580.2004.03304.x

[109] Zhu X Li A Brown B Weiss ER Osawa S Craft CM Mouse cone arrestin expression pattern: light induced translocation in cone photoreceptors. Mol Vis 2002 8 462 71 http://www.molvis.org/molvis/v8/a56/12486395

[110] Newman EA Calcium increases in retinal glial cells evoked by light-induced neuronal activity. J Neurosci 2005 25 5502 10 15944378 10.1523/JNEUROSCI.1354-05.2005 PMC1405916

[111] Galli-Resta L Novelli E Volpini M Strettoi E The spatial organization of cholinergic mosaics in the adult mouse retina. Eur J Neurosci 2000 12 3819 22 11029653 10.1046/j.1460-9568.2000.00280.x

[112] Kinjo TG Szerencsei RT Winkfein RJ Kang K Schnetkamp PP Topology of the retinal cone NCKX2 Na/Ca-K exchanger. Biochemistry 2003 42 2485 91 12600216 10.1021/bi0270788

[113] Berntson AK Morgans CW Distribution of the presynaptic calcium sensors, synaptotagmin I/II and synaptotagmin III, in the goldfish and rodent retinas. J Vis 2003 3 274 80 12803536 10.1167/3.4.3

[114] Ahnelt P Keri C Kolb H Identification of pedicles of putative blue-sensitive cones in the human retina. J Comp Neurol 1990 293 39 53 2312791 10.1002/cne.902930104

[115] Haverkamp S Grunert U Wassle H The synaptic architecture of AMPA receptors at the cone pedicle of the primate retina. J Neurosci 2001 21 2488 500 11264323 10.1523/JNEUROSCI.21-07-02488.2001 PMC6762391

[116] Sterling P Matthews G Structure and function of ribbon synapses. Trends Neurosci 2005 28 20 9 15626493 10.1016/j.tins.2004.11.009

[117] von Gersdorff H Matthews G Electrophysiology of synaptic vesicle cycling. Annu Rev Physiol 1999 61 725 52 10099708 10.1146/annurev.physiol.61.1.725

[118] Baylor DA Nunn BJ Schnapf JL The photocurrent, noise and spectral sensitivity of rods of the monkey Macaca fascicularis. J Physiol 1984 357 575 607 6512705 10.1113/jphysiol.1984.sp015518 PMC1193276

[119] Schnapf JL Nunn BJ Meister M Baylor DA Visual transduction in cones of the monkey Macaca fascicularis. J Physiol 1990 427 681 713 Erratum in: J Physiol Lond1990; 431:757 2100987 10.1113/jphysiol.1990.sp018193 PMC1189952

[120] Laughlin SB de Ruyter van Steveninck RR, Anderson JC. The metabolic cost of neural information. Nat Neurosci 1998 1 36 41 10195106 10.1038/236

[121] Tsukamoto Y Morigiwa K Ueda M Sterling P Microcircuits for night vision in mouse retina. J Neurosci 2001 21 8616 23 11606649 10.1523/JNEUROSCI.21-21-08616.2001 PMC6762784

[122] Missotten L. The ultrastructure of the human retina. Brussels: Arscia Uitgaven; 1965.

[123] Hackenbrock CR Ultrastructural bases for metabolically linked mechanical activity in mitochondria. I. Reversible ultrastructural changes with change in metabolic steady state in isolated liver mitochondria. J Cell Biol 1966 30 269 97 5968972 10.1083/jcb.30.2.269 PMC2107001

[124] Frey TG Renken CW Perkins GA Insight into mitochondrial structure and function from electron tomography. Biochim Biophys Acta 2002 1555 196 203 12206915 10.1016/s0005-2728(02)00278-5

[125] Mannella CA The relevance of mitochondrial membrane topology to mitochondrial function. Biochim Biophys Acta 2006 1762 140 7 16054341 10.1016/j.bbadis.2005.07.001

[126] Mannella CA Pfeiffer DR Bradshaw PC Moraru II Slepchenko B Loew LM Hsieh CE Buttle K Marko M Topology of the mitochondrial inner membrane: dynamics and bioenergetic implications. IUBMB Life 2001 52 93 100 11798041 10.1080/15216540152845885

[127] Renken C Siragusa G Perkins G Washington L Nulton J Salamon P Frey TG A thermodynamic model describing the nature of the crista junction: a structural motif in the mitochondrion. J Struct Biol 2002 138 137 44 12160710 10.1016/s1047-8477(02)00012-6

[128] Gilkerson RW Selker JM Capaldi RA The cristal membrane of mitochondria is the principal site of oxidative phosphorylation. FEBS Lett 2003 546 355 8 12832068 10.1016/s0014-5793(03)00633-1

[129] Issa NP Hudspeth AJ Characterization of fluo-3 labelling of dense bodies at the hair cell's presynaptic active zone. J Neurocytol 1996 25 257 66 8793731 10.1007/BF02284801

[130] Vollrath L Meyer A Buschmann F Ribbon synapses of the mammalian retina contain two types of synaptic bodies--ribbons and spheres. J Neurocytol 1989 18 115 20 2709046 10.1007/BF01188430

[131] Shoshan-Barmatz V Israelson A The voltage-dependent anion channel in endoplasmic/sarcoplasmic reticulum: characterization, modulation and possible function. J Membr Biol 2005 204 57 66 16151701 10.1007/s00232-005-0749-4

[132] Gincel D Vardi N Shoshan-Barmatz V Retinal voltage-dependent anion channel: characterization and cellular localization. Invest Ophthalmol Vis Sci 2002 43 2097 104 12091402

[133] Hahn P Lindsten T Ying GS Bennett J Milam AH Thompson CB Dunaief JL Proapoptotic bcl-2 family members, Bax and Bak, are essential for developmental photoreceptor apoptosis. Invest Ophthalmol Vis Sci 2003 44 3598 605 12882813 10.1167/iovs.02-1113

[134] Cheng EH Sheiko TV Fisher JK Craigen WJ Korsmeyer SJ VDAC2 inhibits BAK activation and mitochondrial apoptosis. Science 2003 301 513 7 12881569 10.1126/science.1083995

[135] Gincel D Silberberg SD Shoshan-Barmatz V Modulation of the Voltage-Dependent Anion Channel (VDAC) by Glutamate. J Bioenerg Biomembr 2000 32 571 83 15254371 10.1023/a:1005670527340

[136] Dzeja PP Terzic A Phosphotransfer networks and cellular energetics. J Exp Biol 2003 206 2039 47 12756286 10.1242/jeb.00426

[137] Johnson CN Adkins NL Georgel P Chromatin remodeling complexes: ATP-dependent machines in action. Biochem Cell Biol 2005 83 405 17 16094444 10.1139/o05-115

[138] Lowry OH Roberts NR Schulz DW Clow JE Clark JR Quantitative histochemistry of retina. II. Enzymes of glucose metabolism. J Biol Chem 1961 236 2813 20 14466982

[139] Rostovtseva TK Bezrukov SM ATP transport through a single mitochondrial channel, VDAC, studied by current fluctuation analysis. Biophys J 1998 74 2365 73 9591663 10.1016/S0006-3495(98)77945-7 PMC1299579

[140] Cortina MS Gordon WC Lukiw WJ Bazan NG Oxidative stress-induced retinal damage up-regulates DNA polymerase gamma and 8-oxoguanine-DNA-glycosylase in photoreceptor synaptic mitochondria. Exp Eye Res 2005 81 742 50 15979612 10.1016/j.exer.2005.04.017

[141] Lohr HR Kuntchithapautham K Sharma AK Rohrer B Multiple, parallel cellular suicide mechanisms participate in photoreceptor cell death. Exp Eye Res 2006 83 380 9 Erratum in: Exp Eye Res. 2006; 83:1522 16626700 10.1016/j.exer.2006.01.014

[142] Forman DS Lynch KJ Smith RS Organelle dynamics in lobster axons: anterograde, retrograde and stationary mitochondria. Brain Res 1987 412 96 106 3607465 10.1016/0006-8993(87)91443-0

[143] Leterrier JF Rusakov DA Nelson BD Linden M Interactions between brain mitochondria and cytoskeleton: evidence for specialized outer membrane domains involved in the association of cytoskeleton-associated proteins to mitochondria in situ and in vitro. Microsc Res Tech 1994 27 233 61 8204913 10.1002/jemt.1070270305

[144] Hollenbeck PJ The pattern and mechanism of mitochondrial transport in axons. Front Biosci 1996 1 d91 102 9159217 10.2741/a118

[145] Bereiter-Hahn J Voth M Metabolic control of shape and structure of mitochondria in situ. Biol Cell 1983 47 309 22

[146] Bloom GS Richards BW Leopold PL Ritchey DM Brady ST GTP gamma S inhibits organelle transport along axonal microtubules. J Cell Biol 1993 120 467 76 7678421 10.1083/jcb.120.2.467 PMC2119514

[147] Chada SR Hollenbeck PJ Nerve growth factor signaling regulates motility and docking of axonal mitochondria. Curr Biol 2004 14 1272 6 15268858 10.1016/j.cub.2004.07.027

[148] Ratner N Bloom GS Brady ST A role for cyclin-dependent kinase(s) in the modulation of fast anterograde axonal transport: effects defined by olomoucine and the APC tumor suppressor protein. J Neurosci 1998 18 7717 26 9742142 10.1523/JNEUROSCI.18-19-07717.1998 PMC6793030

[149] Morfini G Szebenyi G Brown H Pant HC Pigino G DeBoer S Beffert U Brady ST A novel CDK5-dependent pathway for regulating GSK3 activity and kinesin-driven motility in neurons. EMBO J 2004 23 2235 45 15152189 10.1038/sj.emboj.7600237 PMC419914

[150] Pesch UE Fries JE Bette S Kalbacher H Wissinger B Alexander C Kohler K OPA1, the disease gene for autosomal dominant optic atrophy, is specifically expressed in ganglion cells and intrinsic neurons of the retina. Invest Ophthalmol Vis Sci 2004 45 4217 25 15505078 10.1167/iovs.03-1261

[151] Kamei S Chen-Kuo-Chang M Cazevieille C Lenaers G Olichon A Belenguer P Roussignol G Renard N Eybalin M Michelin A Delettre C Brabet P Hamel CP Expression of the Opa1 mitochondrial protein in retinal ganglion cells: its downregulation causes aggregation of the mitochondrial network. Invest Ophthalmol Vis Sci 2005 46 4288 94 16249510 10.1167/iovs.03-1407

[152] Vollrath L Spiwoks-Becker I Plasticity of retinal ribbon synapses. Microsc Res Tech 1996 35 472 87 9016450 10.1002/(SICI)1097-0029(19961215)35:6<472::AID-JEMT6>3.0.CO;2-K

[153] Claes E Seeliger M Michalakis S Biel M Humphries P Haverkamp S Morphological characterization of the retina of the CNGA3(^-/-^)Rho(^-/-^) mutant mouse lacking functional cones and rods. Invest Ophthalmol Vis Sci 2004 45 2039 48 15161873 10.1167/iovs.03-0741

[154] Winkler BS Dang L Malinoski C Easter SS Jr An assessment of rat photoreceptor sensitivity to mitochondrial blockade. Invest Ophthalmol Vis Sci 1997 38 1569 77 9224285

[155] Ponnuswamy A Nulton J Mahaffy JM Salamon P Frey TG Baljon AR Modeling tubular shapes in the inner mitochondrial membrane. Phys Biol 2005 2 73 9 16204859 10.1088/1478-3967/2/1/009

[156] Scorrano L Ashiya M Buttle K Weiler S Oakes SA Mannella CA Korsmeyer SJ A distinct pathway remodels mitochondrial cristae and mobilizes cytochrome c during apoptosis. Dev Cell 2002 2 55 67 11782314 10.1016/s1534-5807(01)00116-2

[157] Fox DA Rubinstein SD Hsu P Developmental lead exposure inhibits adult rat retinal, but not kidney, Na^+^,K(^+^)-ATPase. Toxicol Appl Pharmacol 1991 109 482 93 1649499 10.1016/0041-008x(91)90011-3

[158] Ames A 3rd Li YY Heher EC Kimble CR Energy metabolism of rabbit retina as related to function: high cost of Na^+^ transport. J Neurosci 1992 12 840 53 1312136 10.1523/JNEUROSCI.12-03-00840.1992 PMC6576058

[159] Li Q Lau A Morris TJ Guo L Fordyce CB Stanley EF A syntaxin 1, Galpha(o), and N-type calcium channel complex at a presynaptic nerve terminal: analysis by quantitative immunocolocalization. J Neurosci 2004 24 4070 81 15102922 10.1523/JNEUROSCI.0346-04.2004 PMC6729428

[160] Morgans CW Localization of the alpha(1F) calcium channel subunit in the rat retina. Invest Ophthalmol Vis Sci 2001 42 2414 8 11527958

[161] Morgans CW Bayley PR Oesch NW Ren G Akileswaran L Taylor WR Photoreceptor calcium channels: insight from night blindness. Vis Neurosci 2005 22 561 8 16332266 10.1017/S0952523805225038

[162] Nihira M Anderson K Gorin FA Burns MS Primate rod and cone photoreceptors may differ in glucose accessibility. Invest Ophthalmol Vis Sci 1995 36 1259 70 7775103

[163] Okubo A Sameshima M Unoki K Uehara F Ohba N Ultracytochemical demonstration of glycogen in cone, but not in rod, photoreceptor cells in the rat retina. Ann Anat 1998 180 307 14 9728270 10.1016/S0940-9602(98)80031-9

[164] Kim MH Korogod N Schneggenburger R Ho WK Lee SH Interplay between Na^+^/Ca^2+^ exchangers and mitochondria in Ca^2+^ clearance at the calyx of Held. J Neurosci 2005 25 6057 65 15987935 10.1523/JNEUROSCI.0454-05.2005 PMC6725060

[165] Rottenberg H Marbach M Regulation of Ca^2+^ transport in brain mitochondria. II. The mechanism of the adenine nucleotides enhancement of Ca^2+^ uptake and retention. Biochim Biophys Acta 1990 1016 87 98 2310744 10.1016/0005-2728(90)90010-2

[166] Chalmers S Nicholls DG The relationship between free and total calcium concentrations in the matrix of liver and brain mitochondria. J Biol Chem 2003 278 19062 70 12660243 10.1074/jbc.M212661200

[167] McGrail KM Sweadner KJ Immunofluorescent localization of two different Na,K-ATPases in the rat retina and in identified dissociated retinal cells. J Neurosci 1986 6 1272 83 3012013 10.1523/JNEUROSCI.06-05-01272.1986 PMC6568552

[168] Carafoli E The calcium pumping ATPase of the plasma membrane. Annu Rev Physiol 1991 53 531 47 1828335 10.1146/annurev.ph.53.030191.002531

[169] Lytton J Westlin M Burk SE Shull GE MacLennan DH Functional comparisons between isoforms of the sarcoplasmic or endoplasmic reticulum family of calcium pumps. J Biol Chem 1992 267 14483 9 1385815

[170] Dode L Vilsen B Van Baelen K Wuytack F Clausen JD Andersen JP Dissection of the functional differences between sarco(endo)plasmic reticulum Ca^2+^-ATPase (SERCA) 1 and 3 isoforms by steady-state and transient kinetic analyses. J Biol Chem 2002 277 45579 91 12207029 10.1074/jbc.M207778200

[171] Taylor WR Morgans C Localization and properties of voltage-gated calcium channels in cone photoreceptors of Tupaia belangeri. Vis Neurosci 1998 15 541 52 9685206 10.1017/s0952523898153142

[172] Kreft M Krizaj D Grilc S Zorec R Properties of exocytotic response in vertebrate photoreceptors. J Neurophysiol 2003 90 218 25 12660355 10.1152/jn.01025.2002 PMC2922923

[173] Haeseleer F Imanishi Y Maeda T Possin DE Maeda A Lee A Rieke F Palczewski K Essential role of Ca^2+^-binding protein 4, a Ca_v_1.4 channel regulator, in photoreceptor synaptic function. Nat Neurosci 2004 7 1079 87 15452577 10.1038/nn1320 PMC1352161

[174] Penniston JT Enyedi A Verma AK Adamo HP Filoteo AG Plasma membrane Ca^2+^ pumps. Ann N Y Acad Sci 1997 834 56 64 9405785 10.1111/j.1749-6632.1997.tb52225.x

[175] Guerini D The Ca^2+^ pumps and the Na^+^/Ca^2+^ exchangers. Biometals 1998 11 319 30 10191496 10.1023/a:1009210001608

[176] Duncan JL Yang H Doan T Silverstein RS Murphy GJ Nune G Liu X Copenhagen D Tempel BL Rieke F Krizaj D Scotopic visual signaling in the mouse retina is modulated by high-affinity plasma membrane calcium extrusion. J Neurosci 2006 26 7201 11 16822977 10.1523/JNEUROSCI.5230-05.2006 PMC1987386

[177] Issa NP Hudspeth AJ Clustering of Ca^2+^ channels and Ca(2+)-activated K^+^ channels at fluorescently labeled presynaptic active zones of hair cells. Proc Natl Acad Sci USA 1994 91 7578 82 8052623 10.1073/pnas.91.16.7578 PMC44445

[178] Tucker T Fettiplace R Confocal imaging of calcium microdomains and calcium extrusion in turtle hair cells. Neuron 1995 15 1323 35 8845156 10.1016/0896-6273(95)90011-x

[179] Zenisek D Davila V Wan L Almers W Imaging calcium entry sites and ribbon structures in two presynaptic cells. J Neurosci 2003 23 2538 48 12684438 10.1523/JNEUROSCI.23-07-02538.2003 PMC6742070

[180] McCormack JG Denton RM Mitochondrial Ca^2+^ transport and the role of intramitochondrial Ca^2+^ in the regulation of energy metabolism. Dev Neurosci 1993 15 165 73 7805568 10.1159/000111332

[181] Hansford RG Zorov D Role of mitochondrial calcium transport in the control of substrate oxidation. Mol Cell Biochem 1998 184 359 69 9746330

[182] Brown MR Sullivan PG Geddes JW Synaptic mitochondria are more susceptible to Ca^2+^overload than nonsynaptic mitochondria. J Biol Chem 2006 281 11658 68 16517608 10.1074/jbc.M510303200

[183] Mori N Ishiba Y Kubota S Kobayashi A Higashide T McLaren MJ Inana G Truncation mutation in HRG4 (UNC119) leads to mitochondrial ANT-1-mediated photoreceptor synaptic and retinal degeneration by apoptosis. Invest Ophthalmol Vis Sci 2006 47 1281 92 16565359 10.1167/iovs.05-0493

[184] Ohyama T Hackos DH Frings S Hagen V Kaupp UB Korenbrot JI Fraction of the dark current carried by Ca(2+) through cGMP-gated ion channels of intact rod and cone photoreceptors. J Gen Physiol 2000 116 735 54 11099344 10.1085/jgp.116.6.735 PMC2231818

[185] Migdale K Herr S Klug K Ahmad K Linberg K Sterling P Schein S Two ribbon synaptic units in rod photoreceptors of macaque, human, and cat. J Comp Neurol 2003 455 100 12 12454999 10.1002/cne.10501

[186] Koulen P Kuhn R Wassle H Brandstatter JH Modulation of the intracellular calcium concentration in photoreceptor terminals by a presynaptic metabotropic glutamate receptor. Proc Natl Acad Sci USA 1999 96 9909 14 10449793 10.1073/pnas.96.17.9909 PMC22309

